# Effect of chemically synthesized psilocybin and psychedelic mushroom extract on molecular and metabolic profiles in mouse brain

**DOI:** 10.1038/s41380-024-02477-w

**Published:** 2024-02-20

**Authors:** Orr Shahar, Alexander Botvinnik, Amit Shwartz, Elad Lerer, Peretz Golding, Alex Buko, Ethan Hamid, Dani Kahn, Miles Guralnick, Karin Blakolmer, Gilly Wolf, Amit Lotan, Leonard Lerer, Bernard Lerer, Tzuri Lifschytz

**Affiliations:** 1 Biological Psychiatry Laboratory and Hadassah BrainLabs Center for Psychedelic Research, Hadassah Medical Center, Hebrew University, Jerusalem, Israel; 2 Israel Institute for Biology, Nes Ziona, Israel; 3 Human Metabolome Technologies, Boston, MA USA; 4 Parow Entheobiosciences (ParowBio), Chicago, IL USA; 5 Achva Academic College, Beer Tuvia, Israel; 6 Back of the Yards Algae Sciences (BYAS), Chicago, IL USA

**Keywords:** Neuroscience, Molecular biology

## Abstract

Psilocybin, a naturally occurring, tryptamine alkaloid prodrug, is currently being investigated for the treatment of a range of psychiatric disorders. Preclinical reports suggest that the biological effects of psilocybin-containing mushroom extract or “full spectrum” (psychedelic) mushroom extract (PME), may differ from those of chemically synthesized psilocybin (PSIL). We compared the effects of PME to those of PSIL on the head twitch response (HTR), neuroplasticity-related synaptic proteins and frontal cortex metabolomic profiles in male C57Bl/6j mice. HTR measurement showed similar effects of PSIL and PME over 20 min. Brain specimens (frontal cortex, hippocampus, amygdala, striatum) were assayed for the synaptic proteins, GAP43, PSD95, synaptophysin and SV2A, using western blots. These proteins may serve as indicators of synaptic plasticity. Three days after treatment, there was minimal increase in synaptic proteins. After 11 days, PSIL and PME significantly increased GAP43 in the frontal cortex (*p* = 0.019; *p* = 0.039 respectively) and hippocampus (*p* = 0.015; *p* = 0.027) and synaptophysin in the hippocampus (*p* = 0.041; *p* = 0.05) and amygdala (*p* = 0.035; *p* = 0.004). PSIL increased SV2A in the amygdala (*p* = 0.036) and PME did so in the hippocampus (*p* = 0.014). In the striatum, synaptophysin was increased by PME only (*p* = 0.023). There were no significant effects of PSIL or PME on PSD95 in any brain area when these were analyzed separately. Nested analysis of variance (ANOVA) showed a significant increase in each of the 4 proteins over all brain areas for PME versus vehicle control, while significant PSIL effects were observed only in the hippocampus and amygdala and were limited to PSD95 and SV2A. Metabolomic analyses of the pre-frontal cortex were performed by untargeted polar metabolomics utilizing capillary electrophoresis – Fourier transform mass spectrometry (CE-FTMS) and showed a differential metabolic separation between PME and vehicle groups. The purines guanosine, hypoxanthine and inosine, associated with oxidative stress and energy production pathways, showed a progressive decline from VEH to PSIL to PME. In conclusion, our synaptic protein findings suggest that PME has a more potent and prolonged effect on synaptic plasticity than PSIL. Our metabolomics data support a gradient of effects from inert vehicle via chemical psilocybin to PME further supporting differential effects. Further studies are needed to confirm and extend these findings and to identify the molecules that may be responsible for the enhanced effects of PME as compared to psilocybin alone.

## Introduction

There is renewed interest in studying psychedelics for a number of psychiatric disorders including depression, PTSD, obsessive-compulsive disorders and anxiety [1]. To date, clinical trials have generally been conducted with chemically synthesized psilocybin (PSIL), and little attention has been given to additional, potentially therapeutic, psychoactive or non-psychoactive compounds found in psychedelic mushrooms [2]. The same is true for other psychedelics that are currently a focus of clinical research. Dimethyltryptamine (DMT) is of plant and animal origin and has also been identified in humans [3]. 5-methoxy-dimethyltryptamine (5-MeO-DMT) is derived from the incilius alvarius species of toad [4] and the most common source of mescaline is the peyote cactus [5]. LSD was originally synthesized from lysergic acid which is derived from ergotamine, an alkaloid found in the wheat-infecting ergot fungus [6]. In most clinical studies chemically synthesized forms of these compounds are used. Naturally occurring psychedelic compounds do not exist in isolation but are produced by the organism as part of an extensive milieu that includes molecules of many different types. It is possible that these molecules exert significant biological effects or modulate the action of the active molecule in different ways. In regard to cannabis, it is recognized that not only cannabidiol, but a number of other “entourage” molecules exert significant biological effects along with those of delta-9-tetrahydrocanabinol (Δ -9 THC) and modify the effect of Δ−9 THC [7].

Many experienced psychedelic mushroom users believe that different Psilocybe species have distinctive and characteristic effects [8, 9]. These reports are consistent with observations regarding the effects of, *Inocybe aeruginascens* (high in aeruginascin) as compared to the effects of mushrooms with a high psilocybin and psilocin content. Gartz [10] observed an increased mood-enhancing effect of mushrooms high in aeruginascin as compared to those high in psilocybin, leading him to propose an “entourage effect” of psychedelic mushrooms, whereby additional components of the mushroom extract enhance the effect of psilocybin. A preclinical study compared the effects of an extract from *Psilocybe argentipes* to chemical psilocybin on marble-burying behavior in mice (an animal screening model used to study OCD) [11]. The results of the study showed that the psilocybin mushroom extract was more effective in reducing marble-burying than chemical psilocybin, doing so at a dose of 0.25 mg/kg psilocybin as compared to chemical psilocybin which required 1.5 mg/kg and thus supporting further exploration of an “entourage effect”. Further evidence for a psychedelic mushroom “entourage effect” was provided by Zhuk et al. [12], who showed that *Psilocybe semilanceata* and *Pholiotina cyanopus* mushroom extracts were significantly more potent on in-vivo serotonin 5-HT2A receptor behavioral assays (as demonstrated by the head-twitch response in mice) than the same dose of pure psilocin (the active metabolite of psilocybin).

The variability in effects produced mushroom extracts compared to chemical psilocybin may be explained by differing psilocybin content [13] as well as by the presence and varying levels of other potentially bioactive compounds in the different species of mushrooms. These include tryptamines, such as 4-phosphoryloxy-N-methyltryptamine (baeocystin), 4-phosphoryloxy-N,N,N-trimethyltryptamine (aeruginascin), 4-hydroxy-N-methyltryptamine (norpsilocin) and 4-phosphoryloxytryptamine (norbaeocystin) as well as β-carbolines such as harmine and harmaline, and terpenes [14–18]. Little is known about the effects of these additional components of psychedelic mushrooms in animal models, although there is accumulating evidence regarding the effect of some of the tryptamines of the psilocybin biosynthetic chain. Glatfelter et al. [19] recently showed that only the tertiary amines, psilocybin, psilocin, and 4-acetoxy-N,N-dimethyltryptamine (psilacetin) induced HTR in mice while secondary amines such as baeocystin and norpsilocin and quaternary ammonium compounds such as aeruginascin had little or no effect. It is possible, however, that these tryptamines present in psychedelic mushroom extracts may modulate the effects of psilocybin. In particular, it has been speculated that baeocystin or norpsilocin could potentially contribute to variable subjective effects [20]. Furthermore, additional components of mushroom extract such as beta-carbolines may exert biologically meaningful effects in spite of their low concentrations.

Recent research focusing on the possible therapeutic mechanisms of psychedelic agents has emphasized the putative role of sustained neuroplastic effects [21, 22]. These have been demonstrated by techniques that show an increase in dendritic spines and other microscopic measures of synaptic plasticity induced by psychedelics in cell cultures [23] and in living mouse brain by single photon microscopy [24]. This therapeutic mechanism has received considerable emphasis in the development of novel, non-hallucinogenic, psychedelic agents [25–27].

In this context, it is of considerable mechanistic and translational interest to determine whether PME differs from PSIL in its effects on HTR, induction of neuroplasticity and in the spectrum of metabolic effects that it induces. These questions were the focus of the current study. Recently, we comprehensively characterized HTR induced by PSIL in male C57Bl\6j mice [28]. Whether the characteristics of HTR induced by PME differ from those induced by PSIL under the same experimental conditions and at the same psilocybin dose, is an intriguing open question. A further key question is whether PSIL and PME differ in their effects on neuroplasticity which is increasingly regarded as playing a key role in the beneficial effects of psychedelics on depression and other psychiatric disorders [21, 22]. It is well established that synaptic proteins are pivotally involved in formation, elimination and pruning of synapses and modulation of their strength and plasticity [29]. Synaptic proteins are also involved in synaptic transmission and in maintaining synaptic structure and stability [30]. In accordance with this role, we studied the synaptic proteins GAP43, PSD95, synaptophysin and SV2A. Synaptic proteins may possibly serve markers of synaptic plasticity induced by PSIL and PME although definitive evidence would require demonstration of a correlation with structural plasticity markers. Finally, we performed a metabolomic analysis of the effect of PSIL and PME on the mouse frontal cortex. Metabolomics is an emerging field that seeks to systematically characterize the unique chemical fingerprint that low-molecular-weight molecules leave behind after a given stimulus in a biological specimen [31]. Alterations in biochemical processes such as energy production, oxidative stress and neurotransmission have been linked to various psychiatric disorders including MDD [32]. A recent study analyzed the effects of ayahuasca treatments on primary astrocytes, revealing alterations in various biochemical pathways [33]. We set out to assess cortical metabolomic effects of PSIL and PME in order to identify key pathways that are involved in long-term sustained effects and to potentially identify metabolic pathways that are common to, or differentiate PSIL and PME.

## Methods

### Animals

Experiments were performed on adult (~11 weeks old) C57BL/6J male mice. Sample size was based on prior data. Animals were housed under standardized conditions with a 12-h light/dark cycle, stable temperature (22 ± 1 °C), controlled humidity (55 ± 10%) and free access to food and water. Mice were assigned to experimental groups by randomly extracting them from the holding cages and all experiments were conducted with the investigator blind to treatment assignment. Experiments were conducted in accordance with AAALAC guidelines and were approved by the Authority for Biological and Biomedical Models Hebrew University of Jerusalem, Israel, Animal Care and Use Committee. All efforts were made to minimize animal suffering and the number of animals used.

### Drugs

PSIL was supplied by Usona Institute, (Madison WI, USA) and was determined by AUC at 269.00 nm (UPLC) to contain 98.75 wt. % psilocybin. PME was supplied by Back of the Yards Algae Sciences - Parow Entheobiosciences (Chicago, IL, USA) and was determined to contain psilocybin 1.3 wt.%, psilocin 0.17 wt.%, norpsilocin 0.01 wt.%, baeocystin 0.02 wt.%, norbaeocystin 0.007 wt.% and aerugeniscin 0.008 wt.% by LC-MS/MS (See section, PME cultivation, extraction and analysis, for description of analytic method). PSIL and PME were dissolved in 0.9% NaCl. Whether administered PSIL or PME, mice received psilocybin at a dose of 4.4 mg/kg intraperitoneally (i.p.). The dose of psilocybin that we used (4.4 mg/kg) was chosen on the basis of our previous dose response study on the effect of psilocybin on the head twitch response [28], and in view of the fact this dose is equivalent in mice to a 25 mg dose in humans according to the widely used DoseCal dose conversion methodology [34]. For PME the dose was based on the following calculation: To prepare a solution for a mouse that weighs 30 g so that the mouse will receive 4.4 mg/kg of pure psilocybin, $0.03\,{kg}({since}\,30\,g=0.03\,{kg})\times 4.4\,{mg}/{kg}=0.132\,{{{{{\rm{mg}}}}}}$ of psilocybin per 30 g mouse is needed. Since 1 mg of psilocybin is found in 76.92 mg of PME (1.3%) the amount of PSIL that was given needs to be multiplied by a factor of 76.92, $76.92\times 0.132\,{mg}=10.15\,{{{{{\rm{mg}}}}}}$ of PME is needed per 30 g mouse. In all cases, injections were administered in a standard injection volume of 10 µl/g per mouse. Both PSIL and PME rapidly dissolved completely in saline (0.9% NaCl).

### PME cultivation, extraction and analysis

#### PME cultivation and extraction

*Psilocybe cubensis* was cultivated from genetically confirmed spore material (see www.Entheome.org for whole-genome sequence) germinated on light malt extract agar in a petri dish. Agar-cultured colonies were transferred to sterilized grain jars (rye, white millet, and oats). The colonized grain was transferred into pasteurized bulk substrate bags. The mushrooms were fruited and harvested after 18–28 days. The biomass was dried at 65 °C for 6 h. The dried ground biomass was extracted at a ratio of 1 g of biomass to 20 mL of methanol for 24 h at room temperature in stirred reaction vessels, and filtered through a Büchner funnel whereafter a second extraction took place for a further 24 h. The filtrate from the second extraction was then combined with the filtrate from the first extraction and the methanol was removed using a rotary evaporator. The extract was then suspended in sterile water and spray dried using a nitrogen electrostatic spray dryer (FluidAir, Chicago, Illinois, USA) at conditions of inlet temperature −120 ˚C, outlet temperature −80 ˚C.

#### PME analysis

A Waters Acquity H-Class UPLC and Xevo TQ-S Micro MS and Waters HSS T3 1.8 μm column (2.1 mm × 50 mm) (Waters Corporation, Milford, MA, USA) was used for determining tryptamine concentrations. A seven-point standard curve (10 ppb -1 ppm) was used to calculate concentration from the detector response. It was produced using analytical standards produced by Cerilliant (Round Rock, TX, USA) (psilocybin and psilocin) and Usona (Madison, WI, USA) (norpsilocin, baeocystin, norbaeocystin, and aeruginascin). Values obtained are given in the section, Drugs (above).

### Head twitch response

Head twitch response (HTR) was measured over 20 min by means of a magnetometer apparatus as described by de la Fuente Revenga et al. [35] and Shahar et al. [28]. Briefly, small neodymium magnets (N50, 3 mm diameter × 1 mm height, 50 mg), were attached to the outer ears of mice. After a 5–7-day recovery period, the ear-tagged animals were placed inside a magnetometer apparatus (supplied by Mario de la Fuente Revenga PhD. of Virginia Commonwealth University) immediately after injection of vehicle, PSIL, or PME. The output was amplified (Pyle PP444 phono amplifier) and recorded at 1000 Hz using a NI USB-6001 (National Instruments, US) data acquisition system. Recordings were performed using a MATLAB driver (MathWorks, US, R2021a version, along with the NI myDAQ support package) with the corresponding National Instruments support package for further processing. A custom MATLAB script was used to record the processed signal, which was presented as graphs showing the change in current as peaks (mAh). A custom graphic user interface created in our laboratory was used to further process the recording into an Excel spreadsheet.

### Western blotting

The frontal cortex, amygdala, striatum and hippocampus were dissected and stored at −80 ˚C, lysed in Pierce RIPA sample buffer (Thermo Scientific, USA), supplemented with protease inhibitor cocktail (Roche Diagnostics, Germany) and boiled for 10 min. Equivalent amounts of protein extracts (20 mg) were analyzed by SDS–12% PAGE, followed by transfer of the proteins to polyvinylidene fluoride membrane. Blots were blocked in 5% fat free milk in TBST buffer (Tris-Tween-buffered saline) and incubated in primary antibodies, one hour at room temperature. Primary antibodies included rabbit anti-GAP43 (ab75810, 1:2000; Abcam, UK), rabbit anti-PSD95 (ab238135, 1:2000; Abcam, UK), rabbit anti-Synaptophysin (ab32127, 1:2000; Abcam, UK), rabbit anti-SV2A (ab54351, 1:1000; Abcam, UK) and mouse anti-β-Actin (8H10D10, 1:5000, Cell Signaling Technology). Blots were washed 3 times and incubated with a horseradish peroxidase-conjugated secondary antibodies (1:5000, ABclonal, China) for 1 h, followed by repeated washing with TBST buffer. Proteins were visualized by using enhanced chemiluminescence (ChemiDoc Reader MP, Bio-Rad, USA). The amount of each phosphorylated protein was normalized to the amount of the corresponding total protein detected in the sample and measured by intensity of β-actin. β-actin was selected because recent studies in the field of psychedelics and the utilization of psilocybin have employed Western blot analysis, using beta-actin exclusively as a housekeeping gene [36–39]. β-actin levels did not differ between control group and psilocybin and PME treatment groups (see Supplementary Fig. S37 for graphs). Representative Western blot images from each experimental group for each analysis are shown in Figs. S1–S4.

### Plasma psilocin levels

C57Bl/6j mice were administered PSIL (4.4 mg/kg; *n* = 9) or PME (adjusted to a PSIL dose of 4.4 mg/kg; *n* = 9) by i.p injection. Blood samples were obtained from the mice via submandibular bleeding 15, 30 or 60 min after injection (3 mice in each group) and were collected into 0.5 mL K3EDTA tubes (Greiner). Details of sample processing and HPLC psilocin measurement are given in the Supplementary Information section.

### Metabolomics

Brain tissue (frontal cortex) from mice administered PSIL, PME and VEH (*n* = 5 each), sacrificed 11 days later and stored at −80 ˚C was used for metabolomics assays performed by Human Metabolome Technologies-America (Boston, MA, USA). Brain tissue was placed in a homogenization tube, along with zirconia beads and a solution containing 50% acetonitrile in Milli-Q water (v/v) containing HMT internal standards, after which the sample was homogenized (using beads shaker) at 4 ˚C for 120 s. The homogenate was then centrifuged at 2300 × g, 4 ˚C for 5 min. The upper aqueous layer was centrifugally filtered at 4 ˚C through a 5-kDa cut-off filter. The filtrate was evaporated to dryness under vacuum and reconstituted in Milli-Q water for metabolome analysis. Metabolomic analysis was performed for PSIL, PME and VEH treated mice using untargeted CE-FTMS [40]. Extracted metabolites were measured in the Cation and Anion modes of CE-FTMS based metabolome analysis. An Agilent CE system and Q Exactive Plus mass spectrometer were used for both anionic and cationic measurements. The CE capillary was an uncoated fused silica capillary i.d. 50 μm × 80 cm using HMT running and sheath buffers with mass scan range of m/z 70 to 1050. MetaboAnalyst 5.0 software was used to analyze the data and identify possible associated metabolic pathways and treatment-differentiated metabolic expression patterns.

### Statistical analysis

The experimental data are expressed as the mean ± standard error of the mean (SEM). To determine inter group differences, one- and two-way repeated measure analysis of variance (ANOVA) were used as indicated. Tukey’s or Dunnett’s Multiple Comparison Tests were used to analyze post-hoc comparisons. *p* < 0.05 (two tailed) was the criterion for significance. Samples were excluded from analysis if their value was outside two standard deviations of the mean. Graph Pad Prism, version 9.3.1 software was used for all statistical analyses.

## Results

### Head twitch response

**Fig. 1:**
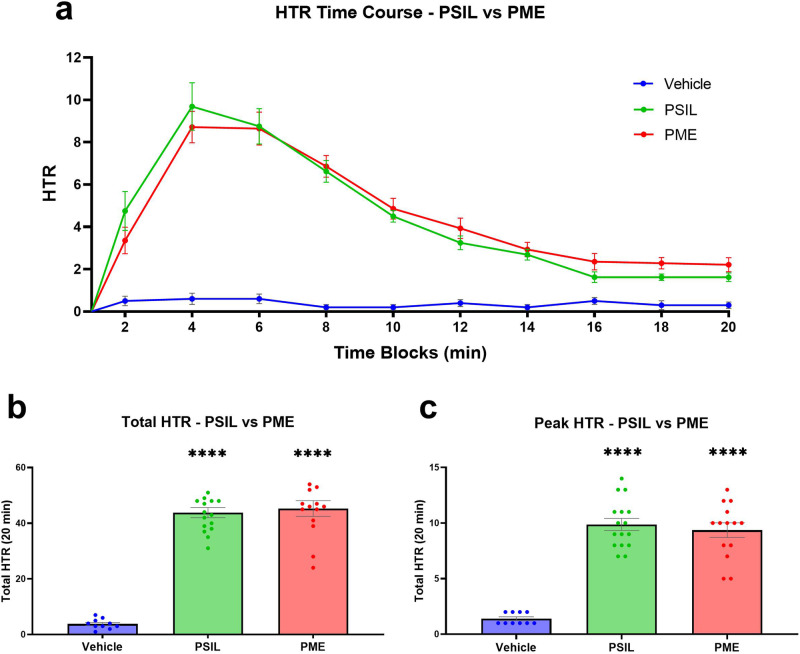
Induction of HTR by PSIL and PME at a psilocybin concentration of 4.4 mg/kg. **a** Time course of the HTR induction by each treatment separated by 2 min time bins. Both treatments induced a peak effect at 4 min post injection. **b** Cumulative HTR during 20 min. **c** The peak HTR effect induced in a 2 min time bin. *n* = 10–16. Compared to vehicle ****p < 0.0001. Error bars represent SEM.

We assessed the effect of PSIL and PME on mouse HTR at a psilocybin dose of 4.4 mg/kg as a representative human clinical trial dose as previously described [28] (Fig. 1). HTR over time (2 min time bins over 20 min) showed a strong increase of HTR (Fig. 1a) compared to vehicle reflected in a between-subject main effect of vehicle vs PSIL/PME in a two-way repeated measure ANOVA (Time × Treatment F [18, 333] = 10.86 *p* < 0.0001, Treatment F [2, 37] = 52.18 *p* < 0.0001), and no difference was observed between PSIL and PME reflected in a within-subject main effect of PSIL vs PME in a two-way repeated measure of ANOVA (Time × Treatment F [9, 252] = 1.162 *p* = 0.3197, Treatment F [1, 28] =  0.04689 *p* = 0.8301). Total and peak PSIL/PME-induced HTR during the 20 min measuring period were compiled (Fig. 1b, c). One-way ANOVA and post hoc Šídák’s analysis of total and peak HTR show a significant difference that both treatments compared to vehicle, with no differences between them (Total HTR: Vehicle vs PSIL/PME *p* < 0.0001, PSIL vs PME *p* = 0.9419. Peak HTR: Vehicle vs PSIL/PME *p* < 0.0001, PSIL vs PME *p* = 0.8662.) These HTR results are for the same mice that participated in the synaptic protein assays. Supplementary Fig. S5 shows HTR results for a larger sample of mice (including those reported above) that were tested for HTR. Similarly, to the previous results, no difference between PSIL and PME was observed.

### Synaptic proteins

**Fig. 2:**
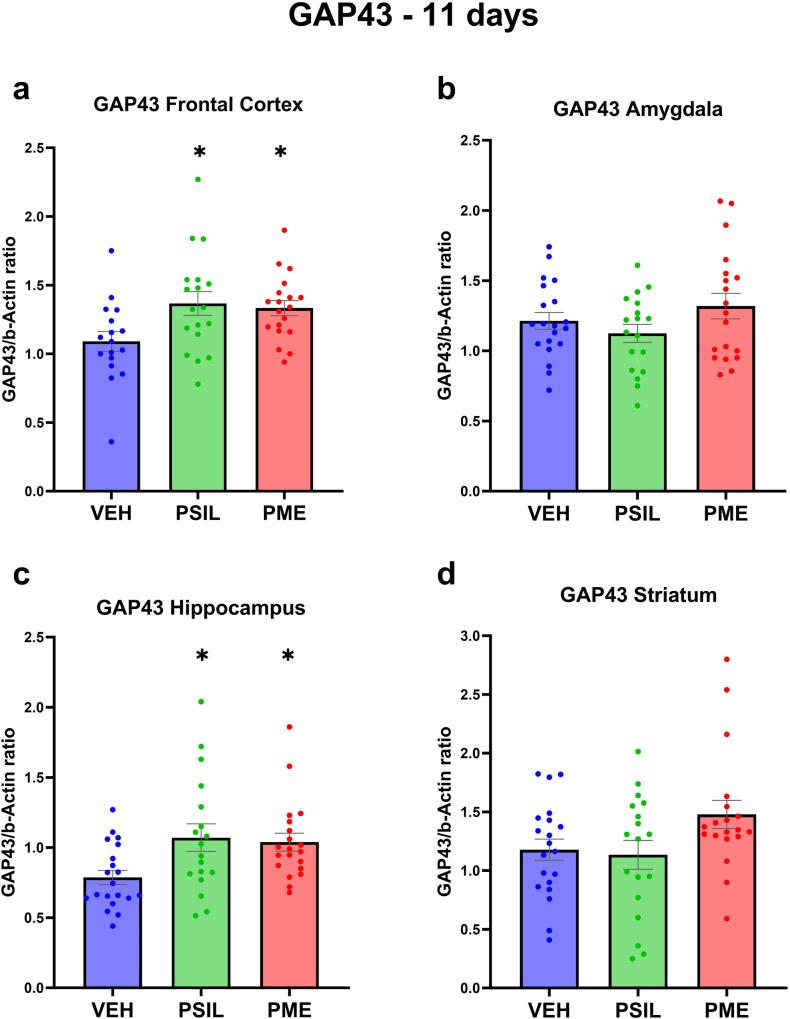
GAP43 levels 11 days after treatment. GAP43 levels 11 days post treatment with VEH, PSIL, or PME in the brain regions of (**a**) Frontal Cortex, (**b**) Amygdala, (**c**) Hippocampus, (**d**) Striatum (*n* = 17–19). One-way ANOVA (**a**) F (2, 51) = 4.250 *p* = 0.0196, (**b**) F (2, 54) = 1.715 *p* = 0.1897, (**c**) F (2, 55) = 4.745 *p* = 0.0126, (**d**) F (2, 54) = 2.832 *p* = 0.0676; Dunnett’s multiple comparisons post hoc test: Compared to VEH, *p < 0.05. Error bars represent SEM.

**Fig. 3:**
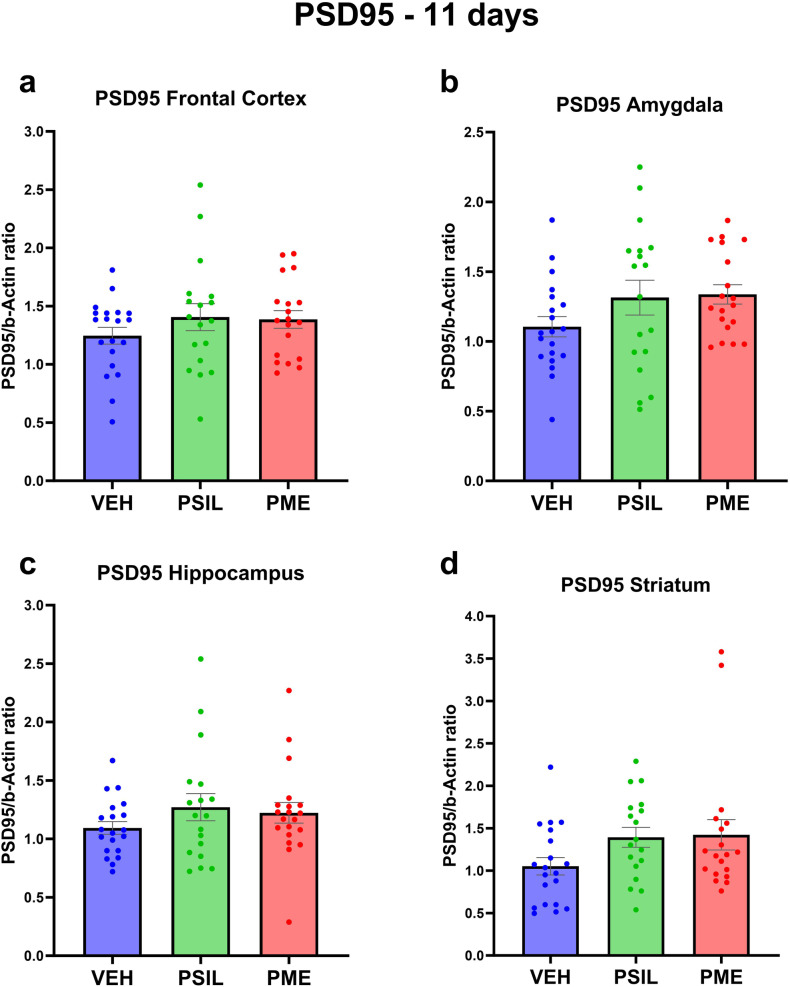
PSD95 levels 11 days after treatment. PSD95 levels 11 days post treatment with VEH, PSIL, or PME in the (**a**) Frontal Cortex, (**b**) Amygdala, (**c**) Hippocampus, (**d**) Striatum (*n* = 19–20). One-way ANOVA (**a**) F (2, 54) = 0.9885 *p* = 0.3788 (**b**) F (2, 54) = 2.046 *p* = 0.1392, (**c**) F (2, 55) = 1.084 *p* = 0.3452, (**d**) F (2, 54) = 2.341 *p* = 0.1059; Dunnett’s multiple comparisons post hoc test. Error bars represent SEM.

**Fig. 4:**
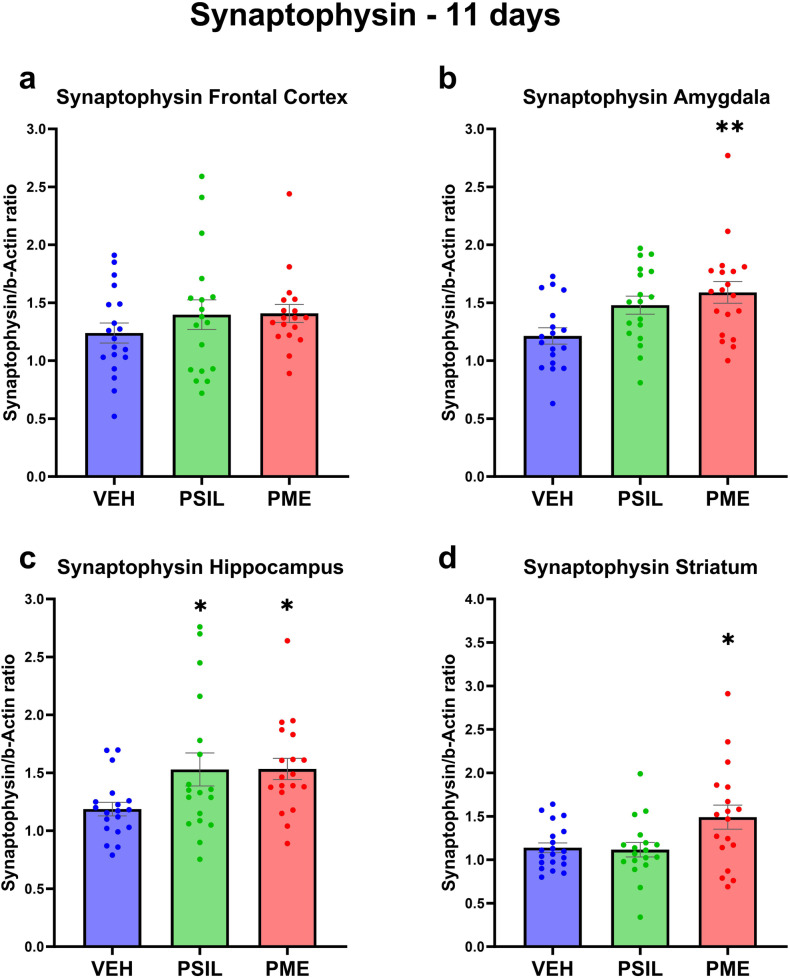
Synaptophysin levels 11 days after treatment. Synaptophysin levels 11 days post treatment in (**a**) Frontal Cortex, (**b**) Amygdala, (**c**) Hippocampus, (**d**) Striatum (*n* = 18–19). One-way ANOVA (**a**) F (2, 52) = 0.9234 *p* = 0.4036, (**b**) F (2, 52) = 5.549 *p* = 0.0065, (**c**) F (2, 53) = 3.842 *p* = 0.0277, (**d**) F (2, 53) = 4.653 *p* = 0.0138; Dunnett’s multiple comparisons post hoc test: Compared to VEH, *p < 0.05, **p < 0.01. Error bars represent SEM.

**Fig. 5:**
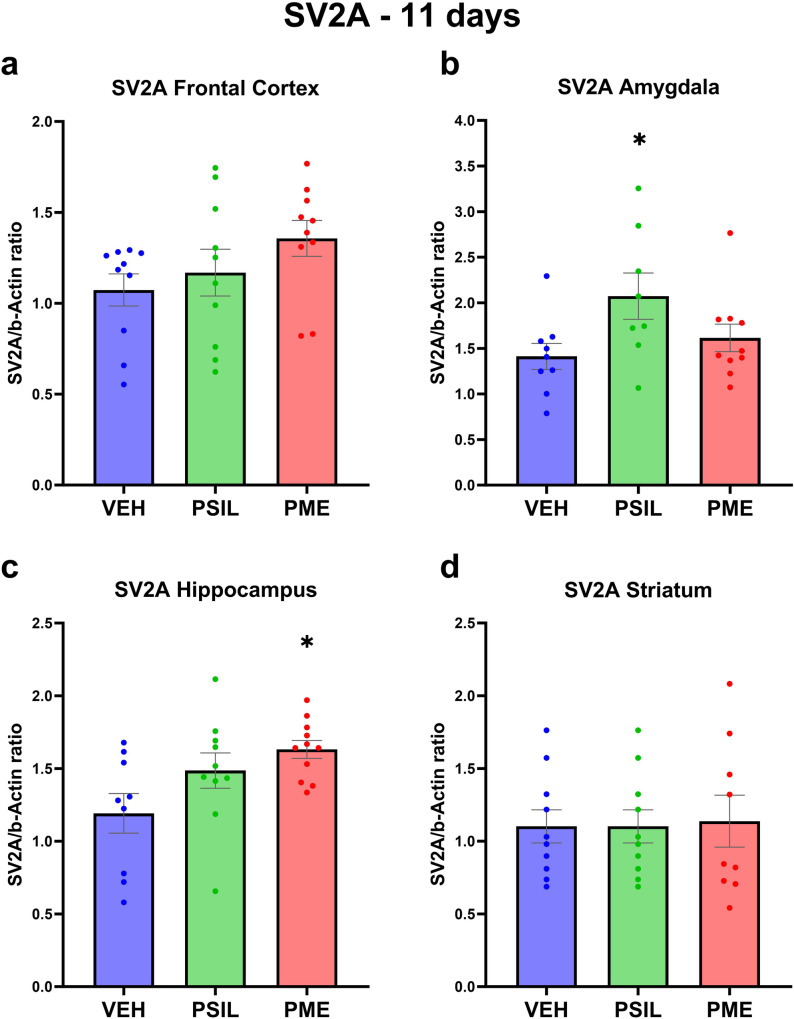
SV2A levels 11 days after treatment. SV2A levels 11 days post treatment with VEH, PSIL, or PME SV2A in (**a**) Frontal Cortex, (**b**) Amygdala, (**c**) Hippocampus, (**d**) Striatum (*n* = 10). One-way ANOVA (**a**) F (2, 27) = 1.844 *p* = 0.1775, (**b**) F (2, 24) = 3.255 *p* = 0.0561, (**c**) F (2, 27) = 4.298 *p* = 0.024, (**d**) F (2, 26) = 0.02239, *p* = 0.9779; Dunnett’s multiple comparisons post hoc test: Compared to VEH, *p < 0.05. Error bars represent SEM.

Using Western blot analysis, we quantified 4 synaptic proteins (GAP43, PSD95, synaptophysin and SV2A – Figs. 2–5) in 4 brain areas (frontal cortex, hippocampus, amygdala and striatum) 11 days after intraperitoneal injection of PSIL and PME, both at 4.4 mg/kg psilocybin. Initially we analyzed each synaptic protein in each brain area separately. We found that PSIL and PME significantly increased GAP43 levels in the frontal cortex (PSIL *p* = 0.019; PME *p* = 0.039) and hippocampus (PSIL *p* = 0.015; PME *p* = 0.027) and synaptophysin levels in the hippocampus (PSIL, *p* = 0.041; PME, *p* = 0.05) and amygdala (PSIL, *p* = 0.035; PME, *p* = 0.004). In the striatum, synaptophysin was increased by PME only (*p* = 0.023). We further observed that PSIL increased SV2A in the amygdala (*p* = 0.036) and PME did so in the hippocampus (*p* = 0.014). There were no significant effects of PSIL or PME on PSD95 in any brain area when these were analyzed separately.

Analyzing the effects of PSIL and PME on synaptic proteins in each of the 4 brain areas separately (Figs S6–S9) we found that hippocampus was the brain area in which the greatest number of significant effects of PSIL and PME were observed. PSIL and PME both significantly increased GAP43 (*p* = 0.015; *p* = 0.027, respectively) and synaptophysin (*p* = 0.041; *p* = 0.035, respectively). PME (*p* = 0.014) but not psilocybin increased SV2A levels. In the amygdala, synaptophysin was increased by PSIL (*p* = 0.035) and PME (*p* = 0.004) and SV2A by PSIL (*p* = 0.036) but not PME while in the frontal cortex GAP43 was significantly increased by both PSIL (*p* = 0.018) and PME (*p* = 0.039). In the striatum, synaptophysin was increased by PME only (*p* = 0.026).

**Fig. 6:**
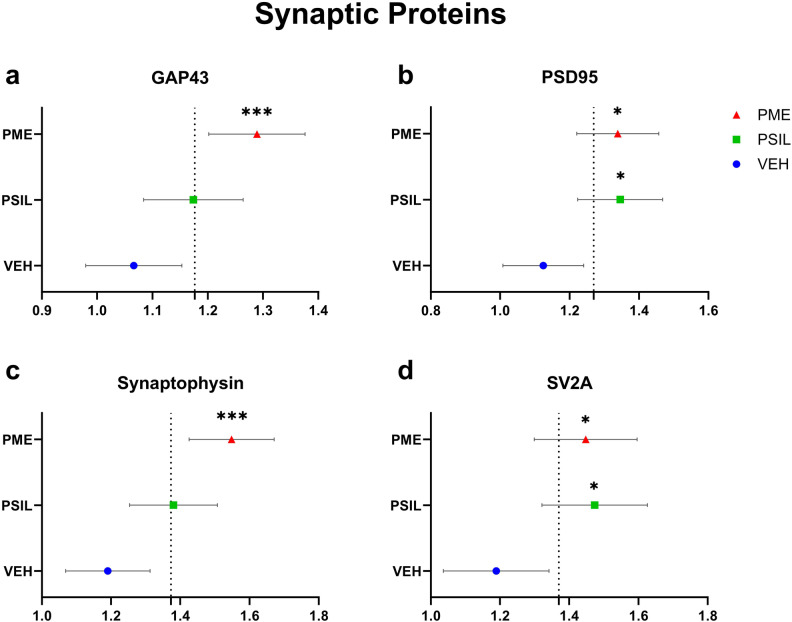
Nested ANOVA of synaptic proteins. Nested ANOVA which compares the overall effect of PSIL, PME and VEH on each synaptic protein separately over all 4 brain areas: (**a**) GAP43 F (2, 223) = 6.354 *p* = 0.0021, (**b**) PSD 95 F (2, 55) = 4.599 *p* = 0.0142, (**c**) Synaptophysin F (2, 56) = 8.563 *p* = 0.0006, (**d**) SV2A F (2, 113) = 4.21 *p* = 0.0172. Compared to VEH, *p < 0.05, **p < 0.01, ***p < 0.001.

A stronger effect of PME on synaptic proteins was highlighted by comparing overall effects of PSIL and PME across all 4 brain areas thus harnessing greater statistical power for the comparison. We performed a nested ANOVA for each synaptic protein separately (Fig. 6a–d). There was a significant overall increase in GAP43 (F [2, 223] = 6.354, *p* = 0.002). PME (*p* = 0.0009) but not PSIL was significant versus vehicle on post hoc testing (Fig. 6a). For PSD95 there was also a significant overall increase across all brain areas (F [2, 55] = 4.599, *p* = 0.014). PSIL (*p* = 0.01) and PME (*p* = 0.022) were both significant versus vehicle on post hoc testing (Fig. 6b). Synaptophysin was also significantly increased across all 4 brain areas (F [2, 56] = 8.563, *p* = 0.0006). On post-hoc testing only PME was significant (*p* = 0.0002) (Fig. 6c). For SV2A the overall effect of treatment was significant across all brain areas (F [2, 113] = 4.21, *p* = 0.017) and both PSIL (*p* = 0.019) and PME (*p* = 0.033) were significant on post-hoc testing (Fig. 6d). Thus, when overall effects across all four brain regions were taken into account, PME increased all 4 synaptic proteins while PSIL increased only 2 of them.

We performed a second nested ANOVA in which effects of PSIL and PME on all 4 synaptic proteins were examined in each brain area separately (Fig. S10a–d). A stronger effect of PME was evident in 3 out of 4 brain areas. In the frontal cortex there was a significant overall effect of treatment (F [2, 57] = 3.579, *p* = 0.034). PME (*p* = 0.028), but not PSIL, was significant versus vehicle on overall testing (Fig. S6a). There was an overall effect of treatment in the hippocampus too (F [2, 58] = 6.083, *p* = 0.004). In this case both PSIL (*p* = 0.005) and PME (*p* = 0.011) were significant (Fig. S6c). In the amygdala (F [2, 56] = 5.273, *p* = 0.008) (Fig. S6b) and striatum (F [2, 55] = 3.516, *p* = 0.034) (Fig. S6d) there was an overall effect of treatment but only PME (*p* = 0.007; *p* = 0.023, respectively) was significant on post-hoc testing.

To determine whether effects on synaptic proteins might be discernable after PSIL and PME administration at an earlier stage than 11 days, we examined levels of three synaptic proteins (GAP43, PSD95 and synaptophysin) in 4 brain areas 3 days after treatment administration (Supplementary Figs. S11–S14). There were significant effects of PSIL (*p* = 0.010) and PME (*p* = 0.037) to increase GAP43 in the striatum. No effects on GAP43 in frontal cortex, hippocampus and amygdala were significant nor effects on PSD95 and synaptophysin in any of the 4 brain areas.

We further examined whether there was any relationship between the HTR induced by PSIL and PME immediately after injection and the effect of these agents on synaptic proteins 11 days later. We compared both total HTR and peak HTR that each compound induced with each of the 4 synaptic proteins in each of the 4 brain areas totaling a total of 64 simple linear regression correlation tests. There was no correlation between synaptic protein increase to any HTR measurement (total or peak HTR) (Supplementary Figs. S15–S30).

### Metabolomics

**Fig. 7:**
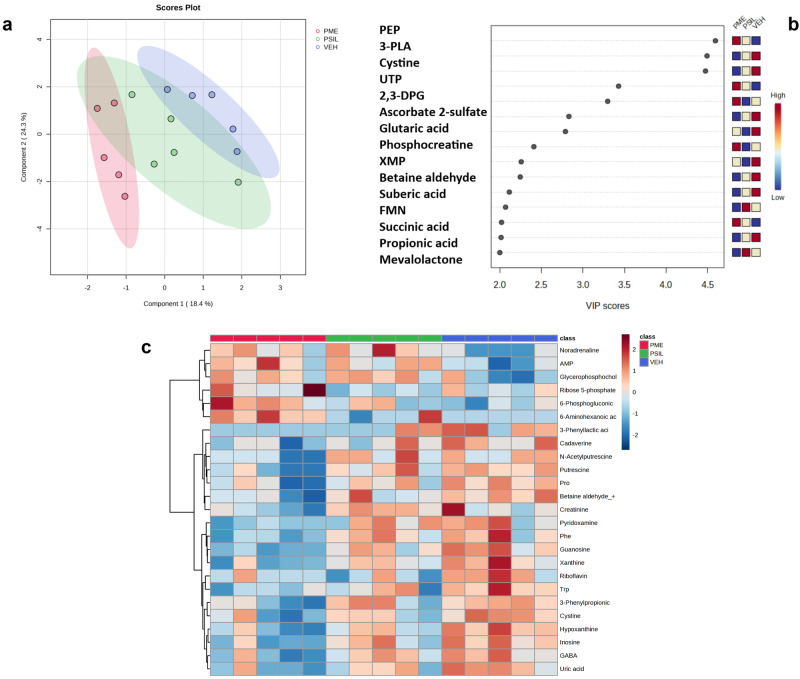
Metabolonic Profiles. **a** PLS-DA plot of the metabolomic profiles of PME (red), which is clearly separated from VEH (blue) and PSIL (green) with partial overlap with both PME and VEH. **b** Variable importance in projection (VIP) scores (>2) - quantifying the contribution of each variable enabling the discrimination between PME, PSIL and VEH groups. Highest VIP scores (VIP > 4.0) were found for 3-Phenyllactic acid, Cystine and Phosphoenolpyruvic acid (PEP). **c** Heatmap of the top 25 differentially expressed metabolites across experimental groups identified by one-way ANOVA (*p* < 0.05). Gradual decreases in metabolite expression levels are visualized moving from vehicle control (right, blue) to psilocybin (PSIL, middle, green) to psychedelic mushroom extract (PME, left, red) groups.

**Fig. 8:**
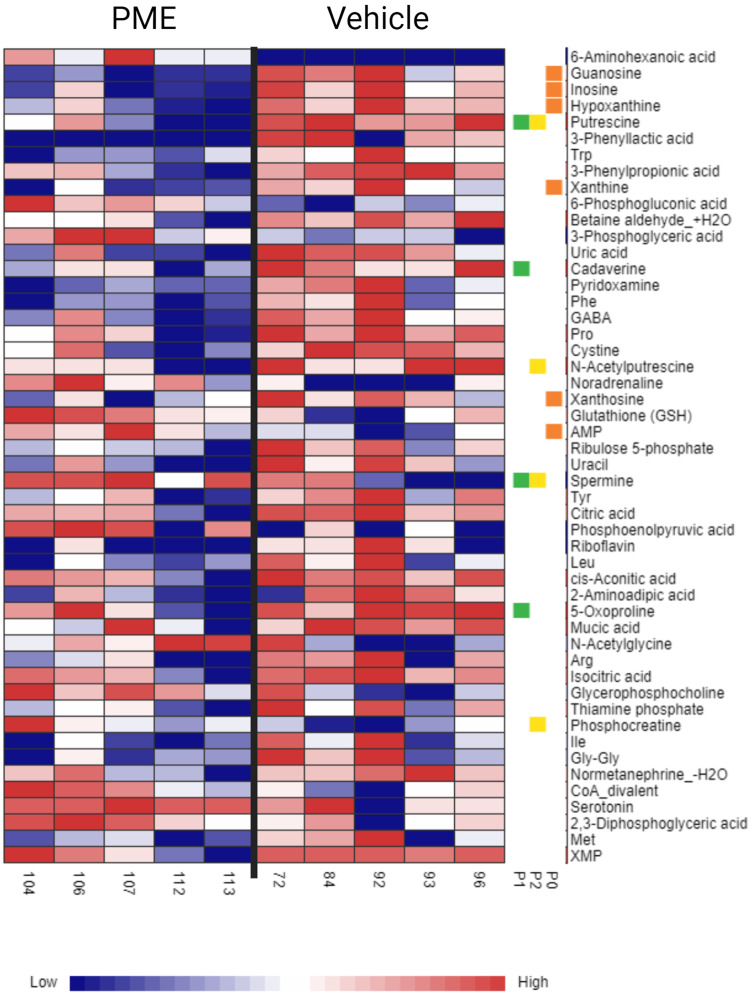
A heatmap visualizing specific pathway analysis of PME and VEH. The significant pathways (FDR < 0.05) and metabolites associated with them are depicted in colored squares: purine metabolism (orange), pyrimidine metabolism (green) and arginine and proline metabolism (yellow).

The CE-FTMS polar method enabled detection of 466 library annotated metabolites, 230 quantitated metabolites and an additional 1566 features with partial identification. An initial unsupervised principal component analysis revealed an overlap between the groups (PME, PSIL and vehicle), with no discernible differences between them (Fig. S31). Therefore, we performed partial least squares-discriminant analysis (PLS-DA), a supervised method which takes into account the predefined groups [41]. The PLS-DA was performed using MetaboAnalyst 5.0 (with 95% confidence interval) to compare the metabolomic profiles between the PME, PSIL, and VEH groups (Fig. 7a). The first two principal components, explaining 18.4% and 24.3% of the variance, were used in the analysis. Components beyond the first two accounted for <10% variance each and were excluded (Fig. S32). The results showed clear separation between PME and VEH but not between PSIL and vehicle nor between PSIL and PME. The principal metabolites contributing to this separation were 3-phenyllactic acid and cystine (under-expressed in the PME group) and phosphoenolpyruvic acid (PEP) (over-expressed in the PME group) (Fig. 7b). In addition, the purines guanosine, hypoxanthine and inosine showed a progressive decline from vehicle to PSIL to PME groups, while their phosphorylated forms are progressively upregulated (Fig. S33). The gradual decrease in expression of key metabolites across the vehicle, PSIL, and PSME groups is visualized in a heatmap (Fig. 7C) showing the top 25 differentially expressed metabolites identified by a one-way ANOVA test comparing all three experimental groups (performed in MetaboAnalyst). Statistical analysis comparing the fold change (1.25 cutoff) between PME vs. VEH revealed many differentiated metabolites (Fig. S34), where guanosine and 6-aminohexanoic acid were found to be significant, withstanding FDR correction (FDR < 0.1). Fig. S35 depicts the fold change comparisons between the other two groups: PME versus PSIL and PSIL versus Vehicle. These analyses did not identify any metabolites with significantly differential expression between the groups after FDR correction at *p* < 0.1. A heatmap visualizing specific pathway analysis, based on the musculus (KEGG) pathway library, between PME and VEH is presented in Fig. 8. The following pathways were found to be significantly differentiated between the groups (Table 1): Purine, pyrimidine, arginine and proline, tryptophan and beta-alanine metabolism. In addition to the unique purine signature, polyamine levels were also significantly altered between vehicle and FSME, while remaining unchanged between PSIL and vehicle. Specifically, putrescine, spermine, and N8-acetylspermidine were significantly modulated in FSME compared to vehicle (*p* < 0.05), but not in PSIL versus vehicle. Relative to vehicle, alpha-synuclein (SNCA) levels, which are promoted by polyamines (Fig. S36), were significantly increased with FSME (*p* < 0.05) but decreased with PSIL administration.

**Table 1:** Significantly differentiated metabolic pathways between PME and VEH.

Pathway	Metabolites	Total	Expected	Hits	*p* Value	FDR
Purine metabolism	Guanosine Inosine Hypoxanthine Xanthine Xanthosine AMP	66	0.705	6	3.30E-05	0.00277
Pyrimidine metabolisms	5-Oxoproline Putrescine Cadaverine Spermine	28	0.299	4	0.000153	0.00641
Arginine and proline metabolism	Putrescine N-Acetylputrescine Spermine Phosophocreatine	38	0.406	4	0.000516	0.0144
Tryptophan metabolism	Spermine Uracil	21	0.224	2	0.02	0.419
beta-Alanine metabolism	Riboflavin	4	0.0427	1	0.0421	0.707

### Plasma psilocin levels

To be certain that observed differences between the effects of PSIL and PME were not due to differences in plasma psilocin, we measured psilocin levels in the plasma of mice administered with these two treatments. Plasma psilocin levels after administration of PSIL 4.4. mg/kg i.p were (mean ± SD) 308.1 ± 29.0 (15 min), 308.9 ± 31.5 (30 min) and 197.0 ± 105.3 (60 min). After PME (PSIL dose 4.4 mg/kg i.p.) plasma psilocin levels were 344.4 ± 79.1 (15 min), 283.7 ± 62.2 (30 min) and 171.9 ± 92.2 (60 min). No differences were statistically significant (Supplementary Fig. S38)

## Discussion

In a comprehensive series of studies that span immediate effects on HTR, changes in synaptic protein levels 3 and 11 days after treatment administration and changes in metabolic parameters at 11 days, we compared the effects of PSIL and PME at the equivalent psilocybin dose. Our findings show no difference in acute effects on HTR. However, we found an effect of PME on synaptic protein levels in 4 brain areas that is significantly more pronounced overall than the effect of PSIL. At the same time point (11 days after treatment administration), the effect of PME on metabolic parameters in the frontal cortex is clearly defined from that of PSIL suggesting a discernibly different or quantitatively stronger therapeutic mechanism.

Our HTR findings contrast with those of Zhuk et al. [12] who compared the effect of psilocin, the active metabolite of psilocybin, on HTR to those of two different psilocin-containing mushroom extracts, *Ph. cyanopus* and *P. semilanceata*. Zhuk et al. [12] found that the two mushroom extracts induced a similar number of HTR compared to pure psilocin, despite the fact that the amount of psilocin present in the extracts was much lower. Zhuk et al. [12] speculated that this phenomenon could be related to the synergistic effect of the other indole alkaloids in the extracts. An important difference between the two studies is that our comparison was based on psilocybin and not psilocin content and the PME and PSIL that we injected were held constant in terms of psilocybin content. Thus, the two studies are not directly comparable.

Our second key focus was on comparing effects of PME and PSIL on key synaptic proteins that we studied as possible markers of neuroplasticity. Neuroplasticity refers to the lifelong capacity of the brain to respond to experiences, learning and the environment and to reorganize structure, function and connections in response to such stimuli. Neuroplastic effects may be defined and measured in structural and functional terms. With regard to the effect of psychedelics, a key focus in the domain of neuroplastic effects has been on structural changes in the synapse with emphasis on growth of new dendrites and proliferation of dendritic spines [1, 23, 24, 42]. It is widely suggested that synaptic plasticity is the central mechanism whereby psychedelics achieve their therapeutic effects [21, 22, 43–45].

Synaptic proteins are pivotally involved in synaptic plasticity by regulating synaptic transmission and because of their role in structural changes that occur during plasticity [46]. Synaptic proteins such as GAP43, PSD95, synaptophysin and SV2A play vital roles in the development, functioning, and plasticity of the nervous system [29]. GAP-43 plays a crucial role in axonal outgrowth, guidance, and pathfinding during development [47, 48]. It has been implicated in the remodeling of synaptic connections, the stabilization of newly formed synapses, and the modulation of neurotransmitter release. Studies have shown that changes in the expression and localization of GAP-43 are associated with synaptic plasticity and learning processes [49, 50]. PSD-95 interacts with various binding partners, such as associated synaptic proteins, ion channels, and cytoskeletal elements, to form a complex network that stabilizes and regulates synaptic function, transmission and formation and maintenance of dendritic spines, receptor trafficking, and synaptic signaling and plasticity mechanisms [51–54]. Synaptophysin is involved in synaptic plasticity [55], and in synaptic vesicle exocytosis [56, 57]. It is an integral component of the synaptic vesicle membrane and interacts with other proteins to regulate vesicle fusion with the presynaptic membrane, thereby facilitating the release of neurotransmitters into the synapse [58–60]. SV2A is involved in multiple aspects of synaptic vesicle function and neurotransmitter release [61]. It plays a crucial role in regulating the trafficking and exocytosis of synaptic vesicles [52, 62]. The absence of SV2A leads to a decrease in neurotransmission mediated by action potentials, while neurotransmission independent of action potentials remains unaffected [62, 63].

Prior evidence that psilocybin or any other psychedelic drugs influence brain levels of the synaptic proteins that we studied, is limited to one report. Raval et al. [64] examined the density of SV2A in pig brain one day and 7 days after a 0.08 mg/kg of psilocybin. SV2A protein density was determined by [3H]UCB-J autoradiography in the hippocampus and the prefrontal cortex. Compared to the saline-treated group, psilocybin treatment was associated with 4.42% higher SV2A in the hippocampus (*p* < 0.0001), one day after psilocybin injection and 9.24% higher SV2A in the hippocampus (*p* = 0.024), seven days after psilocybin. Our results partially overlap with those of Raval et al. [64]. We found that in mice, a dose of PME containing a similar psilocybin dose (adjusted for species differences), increased SV2A in the hippocampus 11 days after administration and a similar dose of PSIL increased SV2A in the striatum, an area that Raval et al. [64] did not examine.

Taking into account both PME and PSIL, it is noteworthy that we found an overall increase in all 4 synaptic proteins examined (GAP43, PSD95, synaptophysin and SV2A) over all 4 brain areas (frontal cortex, hippocampus, amygdala, striatum). The stronger effect of PME was highlighted by the results of the nested analysis of variance which takes into account the effect of the treatments on each of the synaptic proteins over all 4 brain areas and showed that the extract increased all four synaptic proteins, while PSIL increased only two of them. These findings suggest that synaptic protein levels can possibly serve as markers for the effect of psychedelic compounds on synaptic plasticity. Further studies are required to understand regional differences in the effect of PME and PSIL on the synaptic proteins that we studied. It will also be of considerable interest to compare the effect of PME and PSIL on brain derived neurotrophic factor (BDNF) levels. Given the cardinal role of BDNF in supporting growth, survival, and differentiation of both developing and mature neurons and inducing synaptic plasticity, its relationship to psychedelic compounds has been extensively tested in the past years. Animal models have established that BDNF expression is elevated after a single administration of psychedelics [65], resulting in changed neuroplasticity including dendritic complexity, which outlasted the acute effects of the psychedelic. Moreover, chronic psychedelic administrations increased BDNF mRNA and protein levels up to a month after treatment [66–68]. Human studies have usually assessed peripheral plasma BDNF protein levels as a biomarker for neuroplastic modulation, although results are mixed [22]. Most recently, psychedelics have been found to directly bind to the BDNF receptor TrKB, but the therapeutic implications of this interaction are not fully understood [69].

In our metabolomics analyses, partial least squares-discriminant analysis (PLS-DA) revealed complete separation between PME and VEH groups, whereas PSIL components corresponded to a shared area associated with both groups. This initial finding is expected given the natural complexity of biological specimens, as compared to a single chemical molecule, when compared to VEH and may explain the progressive differences in specific metabolite expression. In this context we propose a “purine hypothesis” where “free” purines in their unphosphorylated form show a progressive decline from VEH, to PSIL, to PME while the phosphorylated (bioactive) forms are progressively up-regulated in the counter direction. This may in part explain the energy-dependent biological processes that are attributed to the potentially therapeutic effects of PSIL and PME (anti-inflammatory, neurite outgrowth, neurogenesis, neuroprotection and more) [70]. This system level observation may further demonstrate key longitudinal biotransformation changes (PEP associated ATP and neurotransmitter synthesis, synaptic vesicle formation etc.) that may underlie additional energy supply that enables positive neuroplastic modulations [71]. Furthermore, signaling pathway analysis supported this hypothesis, where the purine metabolism pathway was found to be the most significantly differentiated between PME and VEH. In addition to the unique purine signature, polyamine levels were also significantly altered between vehicle and FSME, while remaining unchanged between PSIL and vehicle. Endogenous polyamines including spermine, spermidine, and putrescine are abundant in the mammalian CNS and the polyamine pathway is intrinsically linked to purine metabolism via methylthioadenosine phosphorylase (MTAP), which plays a key role in both polyamine and adenine salvage pathways. Polyamines are essential for nucleic acid and membrane stability, and are required for cellular growth and differentiation [72]. Exogenous spermine administration rescues age-related neurodegeneration in an autophagy-dependent manner in animal models [73]. However, polyamines also promote alpha-synuclein aggregation and fibrillization [74]. Relative to vehicle, alpha-synuclein (SNCA) levels were increased with FSME but decreased with PSIL administration. While SNCA overexpression is implicated in Parkinson’s pathogenesis basal SNCA is essential for normal synaptic function and integrity during aging [75]. The precise functions of synuclein aggregates in healthy neurons remain unclear. Both SNCA deficiency and excess are deleterious, indicating tight homeostatic control is required. Other metabolic pathways that were found to be significantly different were the pyrimidine metabolism pathway associated with nucleic acid synthesis, and the arginine and proline metabolism pathway associated with metabolism of several amino acids including arginine, ornithine, proline, citrulline, and glutamate in mammals.

We used extract from *Psilocybe cubensis* for our experiment. We cannot exclude that other species of psychedelic mushrooms have different effects as the concentration and ratio of tryptamines and other potentially psychoactive components changes by species, harvest and even part of the same mushroom [76]. Furthermore, storage and processing conditions impact the stability of the individual components [16]. This highlights the need for further identification and refinement of the molecules or combination of compounds that may enhance the activity of psilocybin, in order to ensure consistent results. Furthermore, the extract is not equivalent to a mushroom. Methanolic extraction of mushrooms achieves the highest concentration of the tryptamines, but not all potentially bioactive compounds may be extracted, nor may they be present in equivalent ratios to a those found in mushroom fruiting body. Psilocybe mushrooms also contain water soluble compounds that have been shown to have antioxidant and anti-inflammatory activity, which may have a direct or an indirect effect on neurons and microglia [77].

It is noteworthy that we found no difference in plasma psilocin between mice administered chemical psilocybin and psychedelic mushroom extract containing the same dose of psilocybin. This finding excludes an important potential confounding factor. It is possible that other factors present in mushroom extract could underlie the differences we observed in synaptic protein levels and metabolomics. These include various tryptamines and harmines present in psychedelic mushroom extract. This topic is of considerable interest and is the focus of ongoing studies in our laboratory.

In conclusion, it is imperative to acknowledge certain shortcomings of our study. In recent research in the field of psychedelics western blot analysis has been conducted using whole brain-region homogenates [36–39, 78, 79]. However specific synaptic fractions have been used in some cases such as the study conducted by Berthoux et al. [80] where the focus was on assessing the impact of the synthetic psychedelic, 2,5-Dimethoxy-4-iodoamphetamine (DOI), on glutamatergic transmission and synaptic plasticity in mouse prefrontal cortex (PFC) slices. We recognize that our use of whole brain-region homogenates may introduce variations compared to determinations made with synaptic fractions. Considering this, we plan to address this question in further studies. Additionally, we are aware of the challenge posed by concluding an increase in synaptogenesis based on the 30–50% increases in synaptic proteins observed in our study. Although it is reasonable to suggest that synaptic proteins may serve as markers of synaptic plasticity, this has not been definitively established and more direct approaches to measurement of structural plasticity would enhance our findings. A further shortcoming is the absence of tests reflecting antidepressant-like, anxiogenic-like and other behavioral effects. This is due to the lack of consistency we have encountered in the effects of psilocybin and other psychedelics on behavioral markers of this type. We continue to work on identifying appropriate tests to use in this context. We also note that inclusion of male mice only is a limitation and also the small size of our samples for the metabolomics studies which raise a potential concern of type 1 error.

Despite these shortcomings, we have shown, in a mouse model, clear synaptic protein pattern differences following the administration of PME and PSIL and have demonstrated for the first-time, alterations in cortical metabolic expression patterns between PME and vehicle treated mice and have suggested possible pathway-related mechanisms to explain these differences. While our data do not provide conclusive evidence for the therapeutic superiority of naturally-derived psychedelic mushroom extract over chemical psilocybin, they open the door to serious consideration of the potential of combinations of molecules found in psychedelic mushrooms, especially those related to the psilocybin biosynthetic pathway, with psilocybin. Such combinations may not only have enhanced or more prolonged therapeutic effects but may result in even more effective combinations by increasing the amount of the additional neuroactive compounds that are only present in extremely small amounts naturally.

## Supplementary information

Supplemental Material

## Data Availability

Data will be provided for further analysis to qualified investigators. Please contact lerer@mail.huji.ac.il or tzuri.lifschytz@mail.huji.ac.il

## References

[1] McClure-Begley TD, Roth BL. The promises and perils of psychedelic pharmacology for psychiatry. Nat Rev Drug Discov. 2022;21:463–73. 35301459 10.1038/s41573-022-00421-7

[2] Strauss D, Ghosh S, Murray Z, Gryzenhout M. An overview on the taxonomy, phylogenetics and ecology of the psychedelic genera psilocybe, panaeolus, pluteus and gymnopilus. Front For Glob Change. 2022;5:1–9.

[3] Barker SA. N, N-Dimethyltryptamine (DMT), an endogenous hallucinogen: past, present, and future research to determine its role and function. Front Neurosci. 2018;12:536. 30127713 10.3389/fnins.2018.00536 PMC6088236

[4] Uthaug MV, Lancelotta R, van Oorsouw K, Kuypers KPC, Mason N, Rak J, et al. A single inhalation of vapor from dried toad secretion containing 5-methoxy-N,N-dimethyltryptamine (5-MeO-DMT) in a naturalistic setting is related to sustained enhancement of satisfaction with life, mindfulness-related capacities, and a decrement of psychopathological symptoms. Psychopharmacology. 2019;236:2653–66. 30982127 10.1007/s00213-019-05236-w PMC6695371

[5] Dinis-Oliveira RJ, Pereira CL, da Silva DD. Pharmacokinetic and pharmacodynamic aspects of peyote and mescaline: clinical and forensic repercussions. Curr Mol Pharm. 2019;12:184–94. 10.2174/1874467211666181010154139 PMC6864602 30318013

[6] Schiff PL. Ergot and its alkaloids. Am J Pharm Educ. 2006;70:98. 17149427 10.5688/aj700598 PMC1637017

[7] Ferber SG, Namdar D, Hen-Shoval D, Eger G, Koltai H, Shoval G, et al. The “Entourage Effect”: terpenes coupled with cannabinoids for the treatment of mood disorders and anxiety disorders. Curr Neuropharmacol. 2020;18:87–96. 31481004 10.2174/1570159X17666190903103923 PMC7324885

[8] Jikoms N. Do magic mushrooms have ‘strains’ like cannabis? 2022. https://www.leafly.com/news/science-tech/do-magic-mushrooms-have-strains-like-cannabis. Accessed 27 June 2023.

[9] FreshCap Mushrooms. Do All Magic Mushrooms Have The Same Effect? YouTube. 2023. https://www.youtube.com/watch?v=PBEHe5cC9qg. Accessed 27 June 2023.

[10] Gartz J. Analysis of Aeruginascin in fruit bodies of the mushroom inocybe aeruginascens. Int J Crude Drug Res. 1989;27:141–4.

[11] Matsushima Y, Shirota O, Kikura-Hanajiri R, Goda Y, Eguchi F. Effects of Psilocybe argentipes on marble-burying behavior in mice. Biosci Biotechnol Biochem. 2009;73:1866–8. 19661714 10.1271/bbb.90095

[12] Zhuk O, Jasicka-Misiak I, Poliwoda A, Kazakova A, Godovan VV, Halama M, et al. Research on acute toxicity and the behavioral effects of methanolic extract from psilocybin mushrooms and psilocin in mice. Toxins. 2015;7:1018–29. 25826052 10.3390/toxins7041018 PMC4417952

[13] Van Court RC, Wiseman MS, Meyer KW, Ballhorn DJ, Amses KR, Slot JC, et al. Diversity, biology, and history of psilocybin-containing fungi: suggestions for research and technological development. Fungal Biol. 2022;126:308–19. 35314062 10.1016/j.funbio.2022.01.003

[14] Blei F, Dörner S, Fricke J, Baldeweg F, Trottmann F, Komor A, et al. Simultaneous production of psilocybin and a cocktail of β-carboline monoamine oxidase inhibitors in “Magic” Mushrooms. Chem A Eur J. 2020;26:729–34. 10.1002/chem.201904363 PMC7003923 31729089

[15] Leung AY, Paul AG. Baeocystin and norbaeocystin: new analogs of psilocybin from Psilocybe baeocystis. J Pharm Sci. 1968;57:1667–71. 5684732 10.1002/jps.2600571007

[16] Gotvaldová K, Hájková K, Borovička J, Jurok R, Cihlářová P, Kuchař M. Stability of psilocybin and its four analogs in the biomass of the psychotropic mushroom Psilocybe cubensis. Drug Test Anal. 2021;13:439–46. 33119971 10.1002/dta.2950

[17] Lenz C, Wick J, Hoffmeister D. Identification of ω-N-Methyl-4-hydroxytryptamine (Norpsilocin) as a Psilocybe natural product. J Nat Prod. 2017;80:2835–8. 28929753 10.1021/acs.jnatprod.7b00407

[18] Dörner S, Rogge K, Fricke J, Schäfer T, Wurlitzer JM, Gressler M, et al. Genetic survey of psilocybe natural products. ChemBioChem. 2022;23:e202200249. 35583969 10.1002/cbic.202200249 PMC9400892

[19] Glatfelter GC, Pottie E, Partilla JS, Sherwood AM, Kaylo K, Pham DNK, et al. Structure–activity relationships for psilocybin, baeocystin, aeruginascin, and related analogues to produce pharmacological effects in mice. ACS Pharmacol Transl Sci. 2022;5:1181–96. 36407948 10.1021/acsptsci.2c00177 PMC9667540

[20] Sherwood AM, Halberstadt AL, Klein AK, McCorvy JD, Kaylo KW, Kargbo RB, et al. Synthesis and biological evaluation of tryptamines found in hallucinogenic mushrooms: norbaeocystin, baeocystin, norpsilocin, and aeruginascin. J Nat Prod. 2020;83:461–7. 32077284 10.1021/acs.jnatprod.9b01061

[21] Grieco SF, Castrén E, Knudsen GM, Kwan AC, Olson DE, Zuo Y, et al. Psychedelics and neural plasticity: therapeutic implications. J Neurosci. 2022;42:8439–49. 36351821 10.1523/JNEUROSCI.1121-22.2022 PMC9665925

[22] Calder AE, Hasler G. Towards an understanding of psychedelic-induced neuroplasticity. Neuropsychopharmacology. 2023;48:104–12. 36123427 10.1038/s41386-022-01389-z PMC9700802

[23] Ly C, Greb AC, Cameron LP, Wong JM, Barragan EV, Wilson PC, et al. Psychedelics promote structural and functional neural plasticity. Cell Rep. 2018;23:3170–82. 29898390 10.1016/j.celrep.2018.05.022 PMC6082376

[24] Shao LX, Liao C, Gregg I, Davoudian PA, Savalia NK, Delagarza K, et al. Psilocybin induces rapid and persistent growth of dendritic spines in frontal cortex in vivo. Neuron. 2021;109:2535–44. 10.1016/j.neuron.2021.06.008 PMC8376772 34228959

[25] Cao D, Yu J, Wang H, Luo Z, Liu X, He L, et al. Structure-based discovery of nonhallucinogenic psychedelic analogs. Science. 2022;375:403–11. 35084960 10.1126/science.abl8615

[26] Cameron LP, Tombari RJ, Lu J, Pell AJ, Hurley ZQ, Ehinger Y, et al. A non-hallucinogenic psychedelic analogue with therapeutic potential. Nature. 2021;589:474–9. 33299186 10.1038/s41586-020-3008-z PMC7874389

[27] Kaplan AL, Confair DN, Kim K, Barros-Álvarez X, Rodriguiz RM, Yang Y, et al. Bespoke library docking for 5-HT(2A) receptor agonists with antidepressant activity. Nature. 2022;610:582–91. 36171289 10.1038/s41586-022-05258-z PMC9996387

[28] Shahar O, Botvinnik A, Esh-Zuntz N, Brownstien M, Wolf R, Lotan A, et al. Role of 5-HT2A, 5-HT2C, 5-HT1A and TAAR1 receptors in the head twitch response induced by 5-hydroxytryptophan and psilocybin: translational implications. Int J Mol Sci. 2022;23:14148. 36430623 10.3390/ijms232214148 PMC9698447

[29] Nazir FH, Becker B, Brinkmalm A, Höglund K, Sandelius Å, Bergström P, et al. Expression and secretion of synaptic proteins during stem cell differentiation to cortical neurons. Neurochem Int. 2018;121:38–49. 30342961 10.1016/j.neuint.2018.10.014 PMC6232556

[30] Truckenbrodt S, Viplav A, Jähne S, Vogts A, Denker A, Wildhagen H, et al. Newly produced synaptic vesicle proteins are preferentially used in synaptic transmission. EMBO J. 2018;37:e98044. 29950309 10.15252/embj.201798044 PMC6068464

[31] Courant F, Antignac J-P, Dervilly-Pinel G, Le Bizec B. Basics of mass spectrometry based metabolomics. PROTEOMICS. 2014;14:2369–88. 25168716 10.1002/pmic.201400255

[32] Mandal PK, Gaur S, Roy RG, Samkaria A, Ingole R, Goel A. Schizophrenia, bipolar and major depressive disorders: overview of clinical features, neurotransmitter alterations, pharmacological interventions, and impact of oxidative stress in the disease process. ACS Chem Neurosci. 2022;13:2784–802. 36125113 10.1021/acschemneuro.2c00420

[33] Zandonadi FS, Silva AAR, Melo AA, Ignarro RS, Matos TS, Santos EA, et al. Understanding ayahuasca effects in major depressive disorder treatment through in vitro metabolomics and bioinformatics. Anal Bioanal Chem. 2023;415:1–18. 10.1007/s00216-023-04556-3 36717401

[34] Janhavi P, Divyashree S, Sanjailal K, Muthukumar S. DoseCal: a virtual calculator for dosage conversion between human and different animal species. Arch Physiol Biochem. 2022;128:426–30. 31746232 10.1080/13813455.2019.1687523

[35] de la Fuente Revenga M, Vohra HZ, González-Maeso J. Automated quantification of head-twitch response in mice via ear tag reporter coupled with biphasic detection. J Neurosci methods. 2020;334:108595. 31954738 10.1016/j.jneumeth.2020.108595 PMC7363508

[36] Jefsen OH, Elfving B, Wegener G, Müller HK. Transcriptional regulation in the rat prefrontal cortex and hippocampus after a single administration of psilocybin. J Psychopharmacol. 2021;35:483–93. 33143539 10.1177/0269881120959614

[37] Custodio RJ, Ortiz DM, Lee HJ, Sayson LV, Buctot D, Kim M, et al. 5-HT2CR is as important as 5-HT2AR in inducing hallucinogenic effects in serotonergic compounds. SSRN. 2022;4121838:1–49.

[38] Almeida CAF, Pereira-Junior AA, Rangel JG, Pereira BP, Costa KCM, Bruno V, et al. Ayahuasca, a psychedelic beverage, modulates neuroplasticity induced by ethanol in mice. Behav Brain Res. 2022;416:113546. 34437939 10.1016/j.bbr.2021.113546

[39] Du Y, Li Y, Zhao X, Yao Y, Wang B, Zhang L, et al. Psilocybin facilitates fear extinction in mice by promoting hippocampal neuroplasticity. Chin Med J. 2023;136:2983–92. 37000971 10.1097/CM9.0000000000002647 PMC10752473

[40] Sugishita T, Tokunaga M, Kami K, Terai K, Yamamoto H, Shinohara H, et al. Determination of the minimum sample amount for Capillary Electrophoresis-Fourier Transform Mass Spectrometry (CE-FTMS)-based metabolomics of colorectal cancer biopsies. Biomedicines. 2023;11:1706. 37371800 10.3390/biomedicines11061706 PMC10296550

[41] Ruiz-Perez D, Guan H, Madhivanan P, Mathee K, Narasimhan G. So you think you can PLS-DA? BMC Bioinforma. 2020;21:1–10. 10.1186/s12859-019-3310-7 PMC7724830 33297937

[42] Jones KA, Srivastava DP, Allen JA, Strachan RT, Roth BL, Penzes P. Rapid modulation of spine morphology by the 5-HT2A serotonin receptor through kalirin-7 signaling. Proc Natl Acad Sci. 2009;106:19575–80. 19889983 10.1073/pnas.0905884106 PMC2780750

[43] Slocum ST, DiBerto JF, Roth BL. Molecular insights into psychedelic drug action. J Neurochem. 2022;162:24–38. 34797943 10.1111/jnc.15540

[44] de la Fuente Revenga M, Zhu B, Guevara CA, Naler LB, Saunders JM, Zhou Z, et al. Prolonged epigenomic and synaptic plasticity alterations following single exposure to a psychedelic in mice. Cell Rep. 2021;37:109836. 34686347 10.1016/j.celrep.2021.109836 PMC8582597

[45] Lukasiewicz K, Baker JJ, Zuo Y, Lu J. Serotonergic psychedelics in neural plasticity. Front Mol Neurosci. 2021;14:748359. 34712118 10.3389/fnmol.2021.748359 PMC8545892

[46] Lohmann C, Kessels HW. The developmental stages of synaptic plasticity. J Physiol. 2014;592:13–31. 24144877 10.1113/jphysiol.2012.235119 PMC3903349

[47] Console-Bram LM, Fitzpatrick-McElligott SG, McElligott JG. Distribution of GAP-43 mRNA in the immature and adult cerebellum: a role for GAP-43 in cerebellar development and neuroplasticity. Dev Brain Res. 1996;95:97–106. 8873980 10.1016/0165-3806(96)00079-x

[48] Cantallops I, Routtenberg A. Activity‐dependent regulation of axonal growth: Posttranscriptional control of the GAP‐43 gene by the NMDA receptor in developing hippocampus. J Neurobiol. 1999;41:208–20. 10512978

[49] Snipes G, Chan S, McGuire C, Costello B, Norden J, Freeman J, et al. Evidence for the coidentification of GAP-43, a growth-associated protein, and F1, a plasticity-associated protein. J Neurosci. 1987;7:4066–75. 3694262 10.1523/JNEUROSCI.07-12-04066.1987 PMC6569085

[50] He Q, Dent EW, Meiri KF. Modulation of actin filament behavior by GAP-43 (neuromodulin) is dependent on the phosphorylation status of serine 41, the protein kinase C site. J Neurosci. 1997;17:3515–24. 9133376 10.1523/JNEUROSCI.17-10-03515.1997 PMC6573702

[51] Chen X, Levy JM, Hou A, Winters C, Azzam R, Sousa AA, et al. PSD-95 family MAGUKs are essential for anchoring AMPA and NMDA receptor complexes at the postsynaptic density. Proc Natl Acad Sci. 2015;112:E6983–92. 26604311 10.1073/pnas.1517045112 PMC4687590

[52] Xu T, Bajjalieh SM. SV2 modulates the size of the readily releasable pool of secretory vesicles. Nat cell Biol. 2001;3:691–8. 11483953 10.1038/35087000

[53] Dore K, Malinow R. Elevated PSD-95 blocks ion-flux independent LTD: a potential new role for PSD-95 in synaptic plasticity. Neuroscience. 2021;456:43–9. 32114099 10.1016/j.neuroscience.2020.02.020 PMC7483149

[54] Coley AA, Gao W-J. PSD95: a synaptic protein implicated in schizophrenia or autism? Prog Neuro-Psychopharmacol Biol Psychiatry. 2018;82:187–94. 10.1016/j.pnpbp.2017.11.016 PMC5801047 29169997

[55] Gylys KH, Fein JA, Yang F, Wiley DJ, Miller CA, Cole GM. Synaptic changes in Alzheimer’s disease: increased amyloid-β and gliosis in surviving terminals is accompanied by decreased PSD-95 fluorescence. Am J Pathol. 2004;165:1809–17. 15509549 10.1016/s0002-9440(10)63436-0 PMC1618663

[56] Shibaguchi H, Takemura K, Kan S, Kataoka Y, Kaibara M, Saito N, et al. Role of synaptophysin in exocytotic release of dopamine from Xenopus oocytes injected with rat brain mRNA. Cell Mol Neurobiol. 2000;20:401–8. 10789836 10.1023/A:1007022428041 PMC11537534

[57] Valtorta F, Pennuto M, Bonanomi D, Benfenati F. Synaptophysin: leading actor or walk‐on role in synaptic vesicle exocytosis? Bioessays. 2004;26:445–53. 15057942 10.1002/bies.20012

[58] Alder J, Kanki H, Valtorta F, Greengard P, Poo MM. Overexpression of synaptophysin enhances neurotransmitter secretion at Xenopus neuromuscular synapses. J Neurosci. 1995;15:511–9. 7823159 10.1523/JNEUROSCI.15-01-00511.1995 PMC6578326

[59] Daly C, Ziff EB. Ca2+-dependent formation of a dynamin-synaptophysin complex: potential role in synaptic vesicle endocytosis. J Biol Chem. 2002;277:9010–5. 11779869 10.1074/jbc.M110815200

[60] Horikawa HP, Kneussel M, El Far O, Betz H. Interaction of synaptophysin with the AP-1 adaptor protein γ-adaptin. Mol Cell Neurosci. 2002;21:454–62. 12498786 10.1006/mcne.2002.1191

[61] Bartholome O, Van den Ackerveken P, Sánchez Gil J, de la Brassinne Bonardeaux O, Leprince P, Franzen R, et al. Puzzling out synaptic vesicle 2 family members functions. Front Mol Neurosci. 2017;10:148. 28588450 10.3389/fnmol.2017.00148 PMC5438990

[62] Crowder KM, Gunther JM, Jones TA, Hale BD, Zhang HZ, Peterson MR, et al. Abnormal neurotransmission in mice lacking synaptic vesicle protein 2A (SV2A). Proc Natl Acad Sci. 1999;96:15268–73. 10611374 10.1073/pnas.96.26.15268 PMC24809

[63] Janz R, Goda Y, Geppert M, Missler M, Südhof TC. SV2A and SV2B function as redundant Ca2+ regulators in neurotransmitter release. Neuron. 1999;24:1003–16. 10624962 10.1016/s0896-6273(00)81046-6

[64] Raval NR, Johansen A, Donovan LL, Ros NF, Ozenne B, Hansen HD, et al. A single dose of psilocybin increases synaptic density and decreases 5-HT2A receptor density in the pig brain. Int J Mol Sci. 2021;22:835. 33467676 10.3390/ijms22020835 PMC7830000

[65] Nardai S, László M, Szabó A, Alpár A, Hanics J, Zahola P, et al. N, N-dimethyltryptamine reduces infarct size and improves functional recovery following transient focal brain ischemia in rats. Exp Neurol. 2020;327:113245. 32067950 10.1016/j.expneurol.2020.113245

[66] De Vos CM, Mason NL, Kuypers KP. Psychedelics and neuroplasticity: a systematic review unraveling the biological underpinnings of psychedelics. Front psychiatry. 2021;12:724606. 34566723 10.3389/fpsyt.2021.724606 PMC8461007

[67] Martin DA, Marona-Lewicka D, Nichols DE, Nichols CD. Chronic LSD alters gene expression profiles in the mPFC relevant to schizophrenia. Neuropharmacology. 2014;83:1–8. 24704148 10.1016/j.neuropharm.2014.03.013 PMC4098645

[68] Colaço CS, Alves SS, Nolli LM, Pinheiro WO, de Oliveira DGR, Santos BWL, et al. Toxicity of ayahuasca after 28 days daily exposure and effects on monoamines and brain-derived neurotrophic factor (BDNF) in brain of Wistar rats. Metab Brain Dis. 2020;35:739–51. 32103409 10.1007/s11011-020-00547-w

[69] Moliner R, Girych M, Brunello CA, Kovaleva V, Biojone C, Enkavi G, et al. Psychedelics promote plasticity by directly binding to BDNF receptor TrkB. Nat Neurosci. 2023;26:1032–41. 37280397 10.1038/s41593-023-01316-5 PMC10244169

[70] Kozlowska U, Nichols C, Wiatr K, Figiel M. From psychiatry to neurology: Psychedelics as prospective therapeutics for neurodegenerative disorders. J Neurochem. 2022;162:89–108. 34519052 10.1111/jnc.15509

[71] Ishida A, Noda Y, Ueda T. Synaptic vesicle-bound pyruvate kinase can support vesicular glutamate uptake. Neurochem Res. 2009;34:807–18. 18751889 10.1007/s11064-008-9833-3 PMC2659335

[72] Sagar NA, Tarafdar S, Agarwal S, Tarafdar A, Sharma S. Polyamines: functions, metabolism, and role in human disease management. Med Sci. 2021;9:44. 10.3390/medsci9020044 PMC8293435 34207607

[73] Xu T-T, Li H, Dai Z, Lau GK, Li B-Y, Zhu W-L, et al. Spermidine and spermine delay brain aging by inducing autophagy in SAMP8 mice. Aging. 2020;12:6401. 32268299 10.18632/aging.103035 PMC7185103

[74] Fernandez CO, Hoyer W, Zweckstetter M, Jares‐Erijman EA, Subramaniam V, Griesinger C, et al. NMR of α‐synuclein–polyamine complexes elucidates the mechanism and kinetics of induced aggregation. EMBO J. 2004;23:2039–46. 15103328 10.1038/sj.emboj.7600211 PMC424375

[75] Calabresi P, Mechelli A, Natale G, Volpicelli-Daley L, Di Lazzaro G, Ghiglieri V. Alpha-synuclein in Parkinson’s disease and other synucleinopathies: from overt neurodegeneration back to early synaptic dysfunction. Cell Death Dis. 2023;14:176. 36859484 10.1038/s41419-023-05672-9 PMC9977911

[76] Gotvaldová K, Borovička J, Hájková K, Cihlářová P, Rockefeller A, Kuchař M. Extensive collection of psychotropic mushrooms with determination of their tryptamine alkaloids. Int J Mol Sci. 2022;23:14068. 36430546 10.3390/ijms232214068 PMC9693126

[77] Nkadimeng SM, Nabatanzi A, Steinmann CM, Eloff JN. Phytochemical, cytotoxicity, antioxidant and anti-inflammatory effects of Psilocybe natalensis magic mushroom. Plants. 2020;9:1127. 32878164 10.3390/plants9091127 PMC7570254

[78] Zanikov T, Gerasymchuk M, Ghasemi Gojani E, Robinson GI, Asghari S, Groves A, et al. The effect of combined treatment of psilocybin and eugenol on lipopolysaccharide-induced brain inflammation in mice. Molecules. 2023;28:2624. 36985596 10.3390/molecules28062624 PMC10056123

[79] Ignjatović Đ, Tovilović-Kovačević G, Mićić B, Tomić M, Djordjevic A, Macut D, et al. Effects of early life overnutrition and hyperandrogenism on spatial learning and memory in a rat model of polycystic ovary syndrome. Hormones Behav. 2023;153:105392. 10.1016/j.yhbeh.2023.105392 37295324

[80] Berthoux C, Barre A, Bockaert J, Marin P, Bécamel C. Sustained activation of postsynaptic 5-HT2A Receptors Gates Plasticity at Prefrontal Cortex Synapses. Cereb Cortex. 2018;29:1659–69. 10.1093/cercor/bhy064 29917056

