# Supplementary material for: Effect of chemically synthesized psilocybin and psychedelic mushroom extract on molecular and metabolic profiles in mouse brain
Source: Mol Psychiatry. 2024 Feb 20;29(7):2059–73. doi: 10.1038/s41380-024-02477-w (PMC11408259; doi:10.1038/s41380-024-02477-w)
Supplement: Supplementary file 1 — Supplemental Material [file 41380_2024_2477_MOESM1_ESM.pdf]

Shahar et al: SUPPLEMENTARY INFORMATION

Sample preparation and HPLC method for plasma psilocin determination

Samples obtained from mice were immediately centrifuged at 4000rpm at 4°C for 15 minutes. The plasma fraction was separated and stabilized with a stock solution of L-ascorbic acid (Merck) 250mM to a final concentration of 25mM. An equivalent volume of acetonitrile (Merck) was added to the stabilized sample to precipitate plasma proteins. The samples were then centrifuged at 10000rpm at 4°C for 10 minutes and the supernatant was separated for HPLC analysis. Samples were kept on ice throughout the entire process until HPLC analysis. Samples were held on wet ice throughout processing until HPLC analysis.

Analysis was conducted on an Agilent 1260 Infinity II HPLC system equipped with a multi-wavelength detector and a Zorbax 300SB-C18, 5uM, 4.6 x 150mm column (Agilent). All HPLC mobile phase reagents were HPLC grade.
Sample Injection volume: 20uL.
Mobile Phase A: 0.1% trifluoracetic acid in water.
Mobile Phase B: Acetonitrile
Flow Rate: 0.5mL/min
0-1min: 100% A
1-21min: Gradient to 100% B
21-25min: 100% B
25-26min: Gradient to 100% A
26-32min: 100% A

Column temperature: 30°C

Detection Wavelength: 260nm

A calibration curve was created using standard dilutions of psilocin from 1000ng/mL to 34ng/mL. The psilocin (Usona Institute) for the calibration standards was dissolved in 1:1 v/v acetonitrile:water with 12.5mM L-ascorbic acid.

Analyte concentrations in the plasma samples were determined using the area under the curve (AUC) of analytes relative to external standards. The calibration curve was created using a linear

model with R^2 = 0.999. Sample psilocin concentration was quantified using the OpenLab Data Analysis Software using the ChemStation integrator.

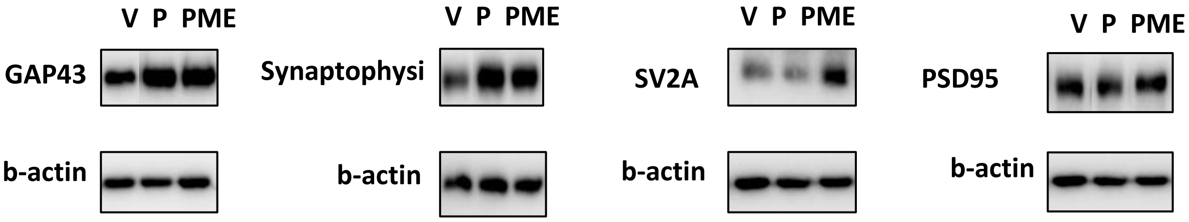

Fig. S1. Representative immunoblots of GAP43, Synaptophysin, SV2A, PSD95 and b-actin protein expression in the hippocampus after V (Vehicle), P (Psilocybin) and PME (psychedelic mushroom

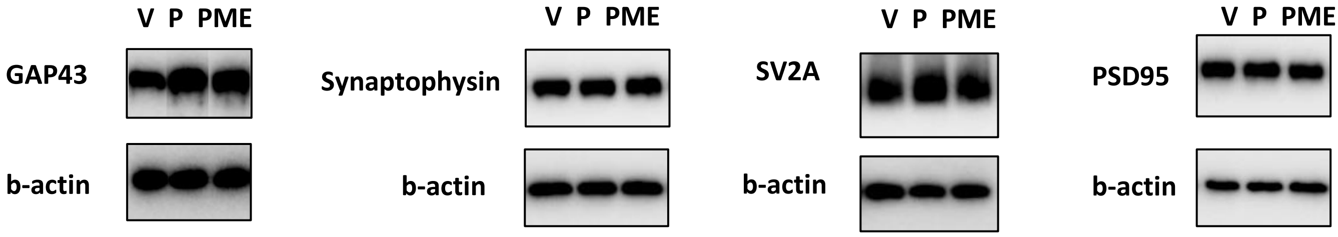

Fig. S2. Representative immunoblots of GAP43, Synaptophysin, SV2A, PSD95 and b-actin protein expression in the frontal cortex after V (Vehicle), P (Psilocybin) and PME (psychedelic mushroom extract) treatments. .

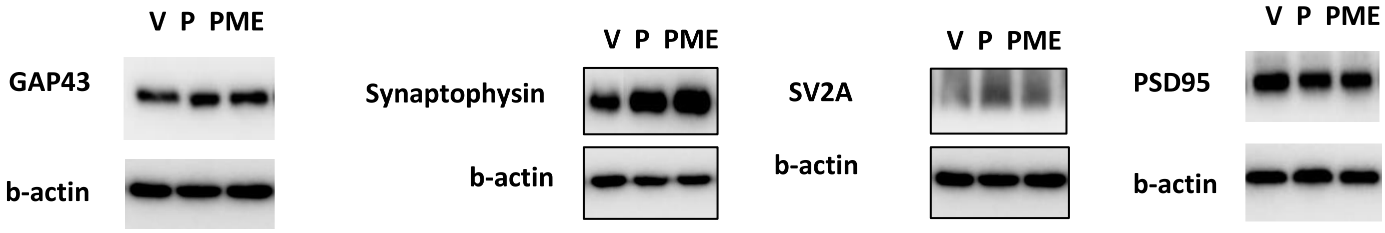

Fig. S3. Representative immunoblots of GAP43, Synaptophysin, SV2A, PSD95 and b-actin protein expression in the amygdala after V (Vehicle), P (Psilocybin) and PME (psychedelic mushroom extract) treatments. .

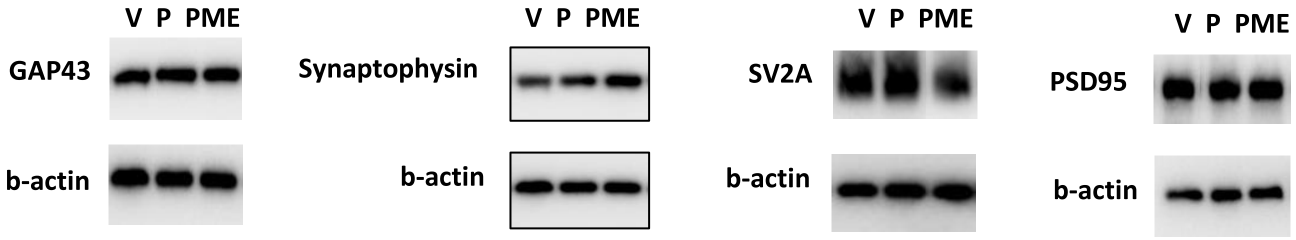

Fig. S4. Representative immunoblots of GAP43, Synaptophysin, SV2A, PSD95 and b-actin protein expression in the striatum after V (Vehicle), P (Psilocybin) and PME (psychedelic mushroom extract) treatments.

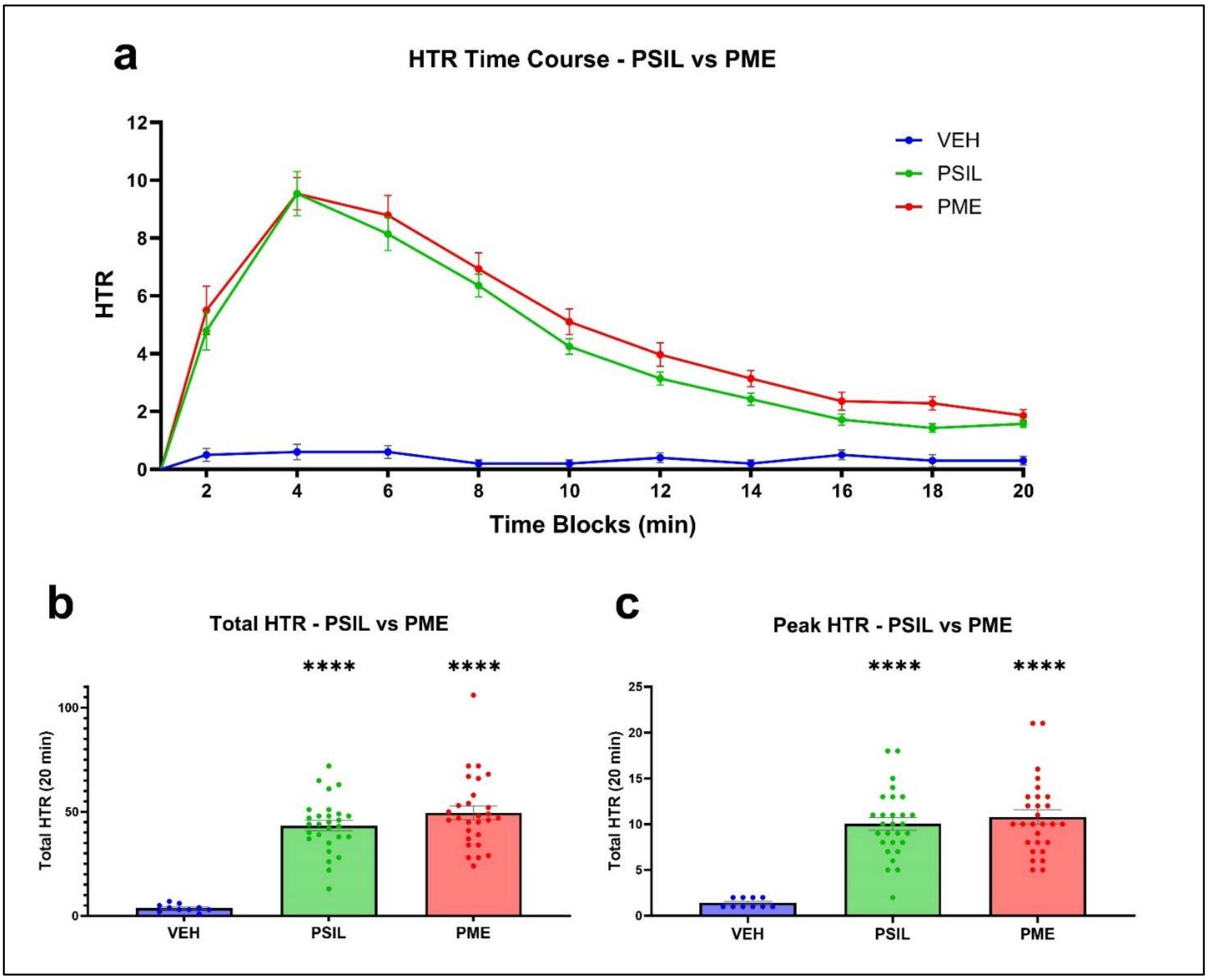

Fig. S5: Induction of HTR by PSIL and PME at a psilocybin concentration of 4.4 mg/kg. (a) Time course of the HTR induction by each treatment separated by 2min time bins. Both treatments induced a peak effect at 4 min post injection. (b) Cumulative HTR during 20 min. (c) The peak HTR effect induced in a 2min time bin. n = 10-28. Compared to vehicle **** $p < 0.0001$. Error bars represent SEM.

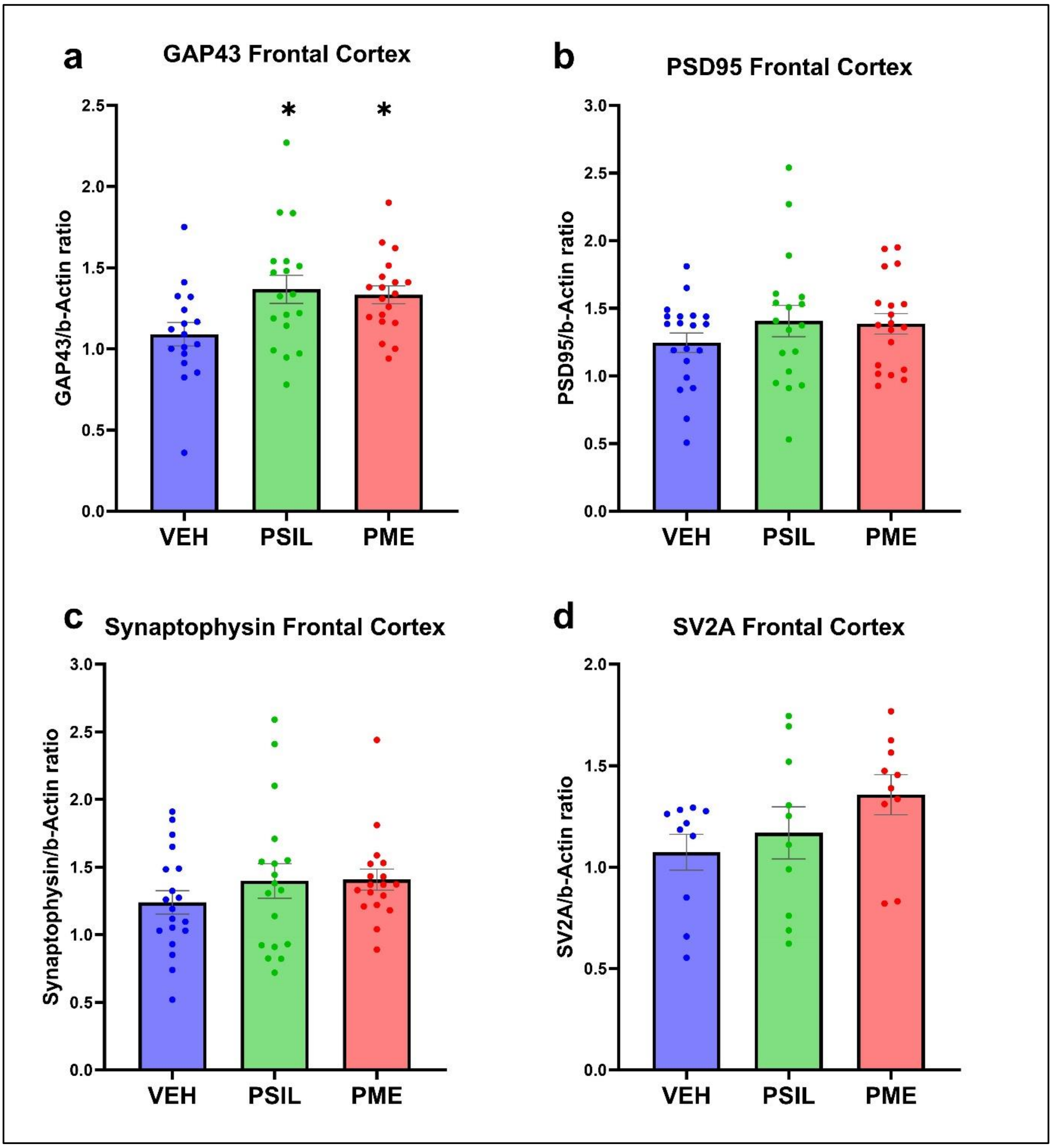

Fig. S6: 11 days post treatment of either VEH, PSIL, or PME Frontal Cortex synaptic protein increase across all synaptic proteins of (a) GAP43 F (2, 51) = 4.250 *p* = 0.0196, (b) PSD95 F (2, 54) = 0.9885 *p* = 0.3788, (c) Synaptophysin F (2, 52) = 0.9234 *p* = 0.4036, (d) SV2A F (2, 27) = 1.844 *p* = 0.1775 (n = 10 - 20). One-way ANOVA, Dunnett's multiple comparisons post hoc test. Compared to VEH, * *p* < 0.05. Compared to VEH, * *p* < 0.05. Error bars represent SEM.

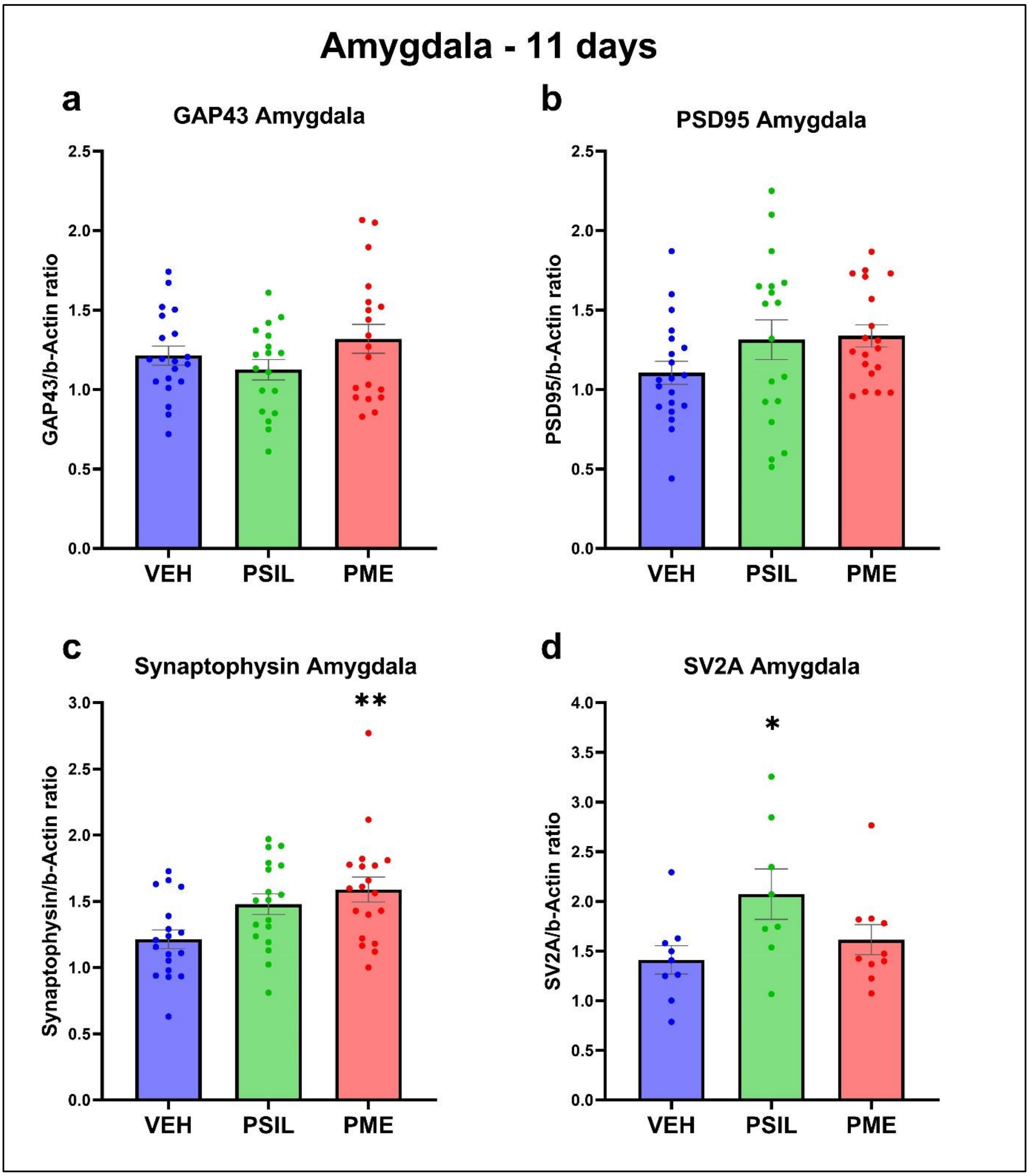

Fig. S7: 11 days post treatment of either VEH, PSIL, or PME Amygdala synaptic protein increase across all synaptic proteins of (a) GAP43 $F (2, 54) = 1.715$ p =0.1897, (b) PSD95 $F (2, 54) = 2.046$ p = 0.1392, (c) Synaptophysin $F (2, 52) = 5.549$ p = 0.0065, (d) SV2A $F (2, 24) = 3.255$ p = 0.0561 (n = 10 - 20). One-way ANOVA, Dunnett's multiple comparisons post hoc test. Compared to VEH, * $p < 0.05$, ** $p < 0.01$. Error bars represent SEM.

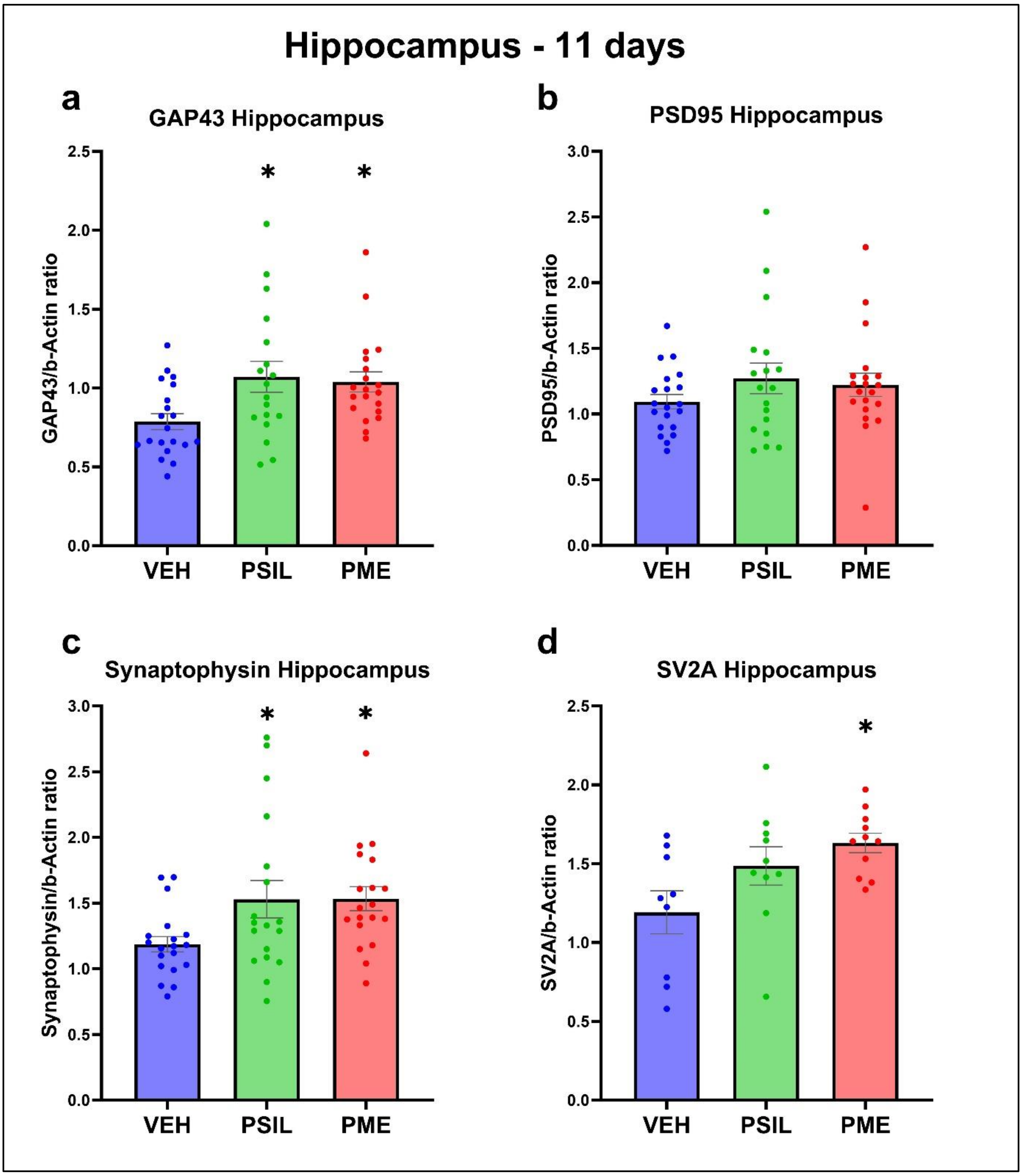

Fig. S8: 11 days post treatment of either VEH, PSIL, or PME Hippocampus synaptic protein increase across all synaptic proteins of (a) GAP43 $F (2, 55) = 4.745$ $p = 0.0126$, (b) PSD95 $F (2, 55) = 1.084$ $p = 0.3452$, (c) Synaptophysin $F (2, 53) = 3.842$ $p = 0.0277$, (d) SV2A $F (2, 27) = 4.298$ $p = 0.024$ (n = 10 - 20). One-way ANOVA, Dunnett's multiple comparisons post hoc test. Compared to VEH, * $p < 0.05$. Error bars represent SEM.

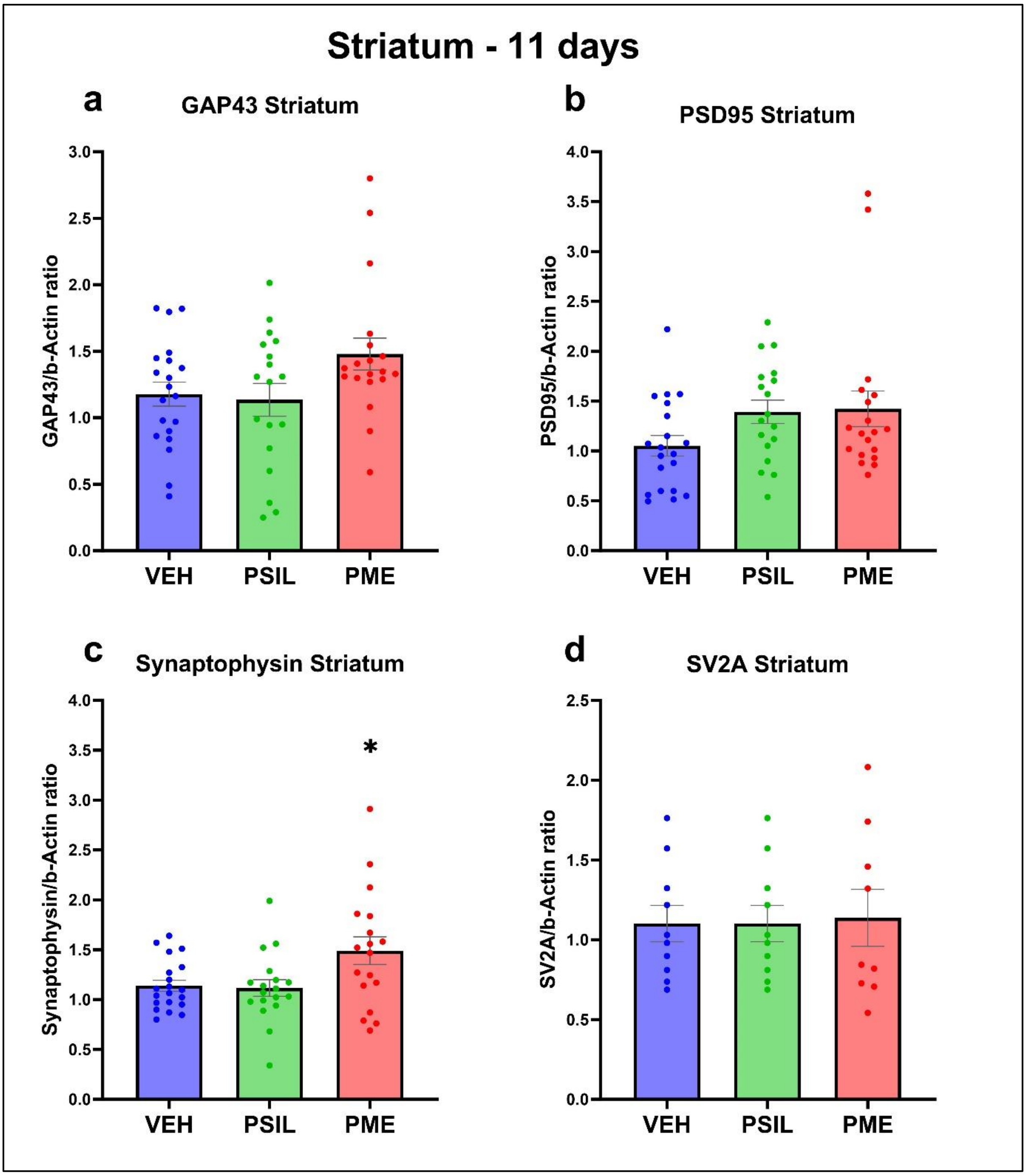

Fig. S9: 11 days post treatment of either VEH, PSIL, or PME Striatum synaptic protein increase across all synaptic proteins of (a) GAP43 $F (2, 54) = 2.832$ $p = 0.0676$, (b) PSD95 $F (2, 54) = 2.341$ $p = 0.1059$, (c) Synaptophysin $F (2, 53) = 4.653$ $p = 0.0138$, (d) SV2A $F (2, 26) = 0.02239$, $p = 0.9779$ ($n = 10 - 20$). One-way ANOVA, Dunnett's multiple comparisons post hoc test. Compared to VEH, * $p < 0.05$. Error bars represent SEM.

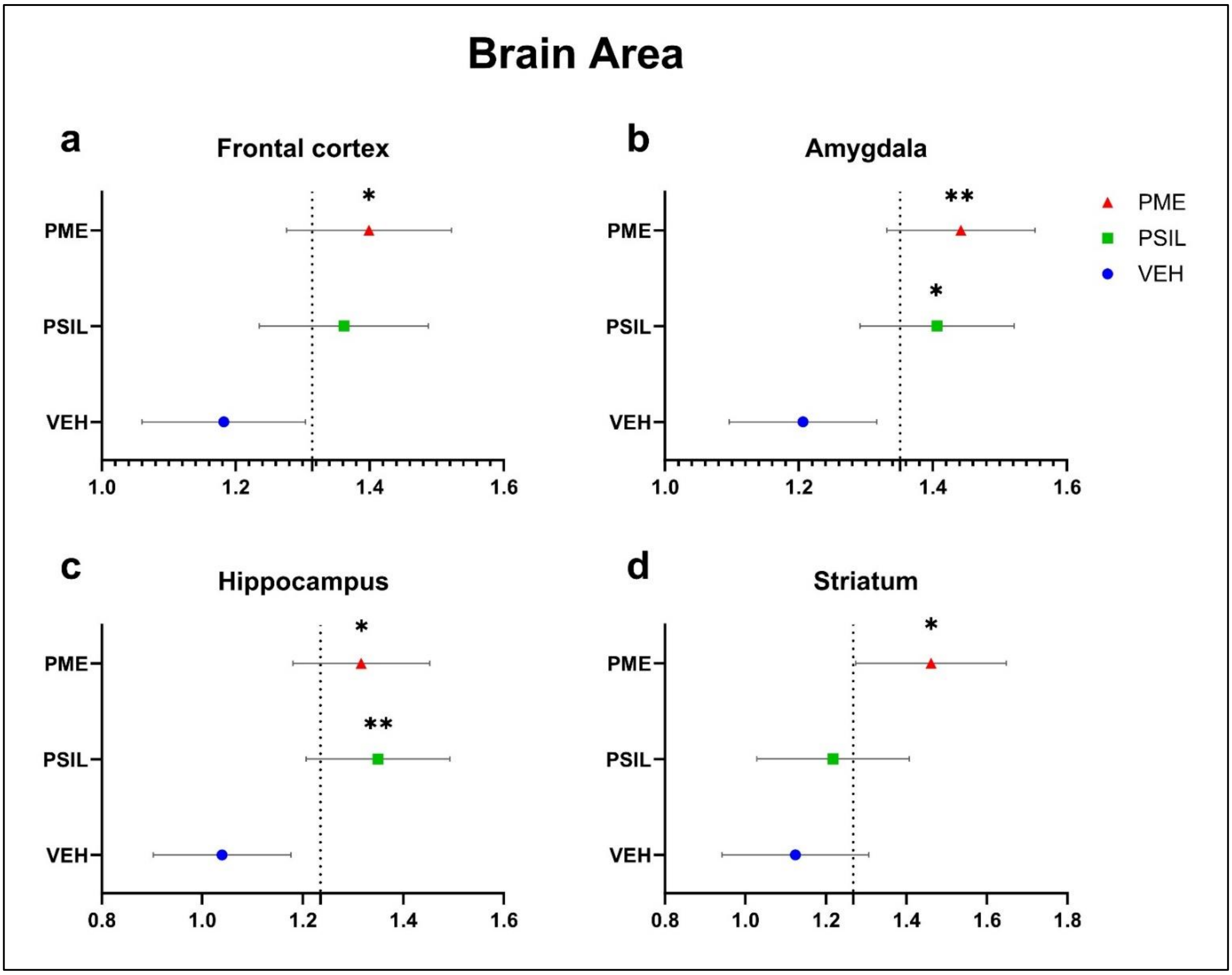

Fig. S10: Nested ANOVA which compares the overall effect of PSIL, PME and VEH on all 4 synaptic proteins in each brain area separately. (a) Frontal Cortex F (2, 57) = 3.579 p = 0.0343, (b) Amygdala F (2, 56) = 5.273 p = 0.008, (c) Hippocampus F (2, 58) = 6.083 p = 0.004, (d) Striatum F (2, 55) = 3.516 p = 0.0366. Compared to VEH, * $p < 0.05$, ** $p < 0.01$

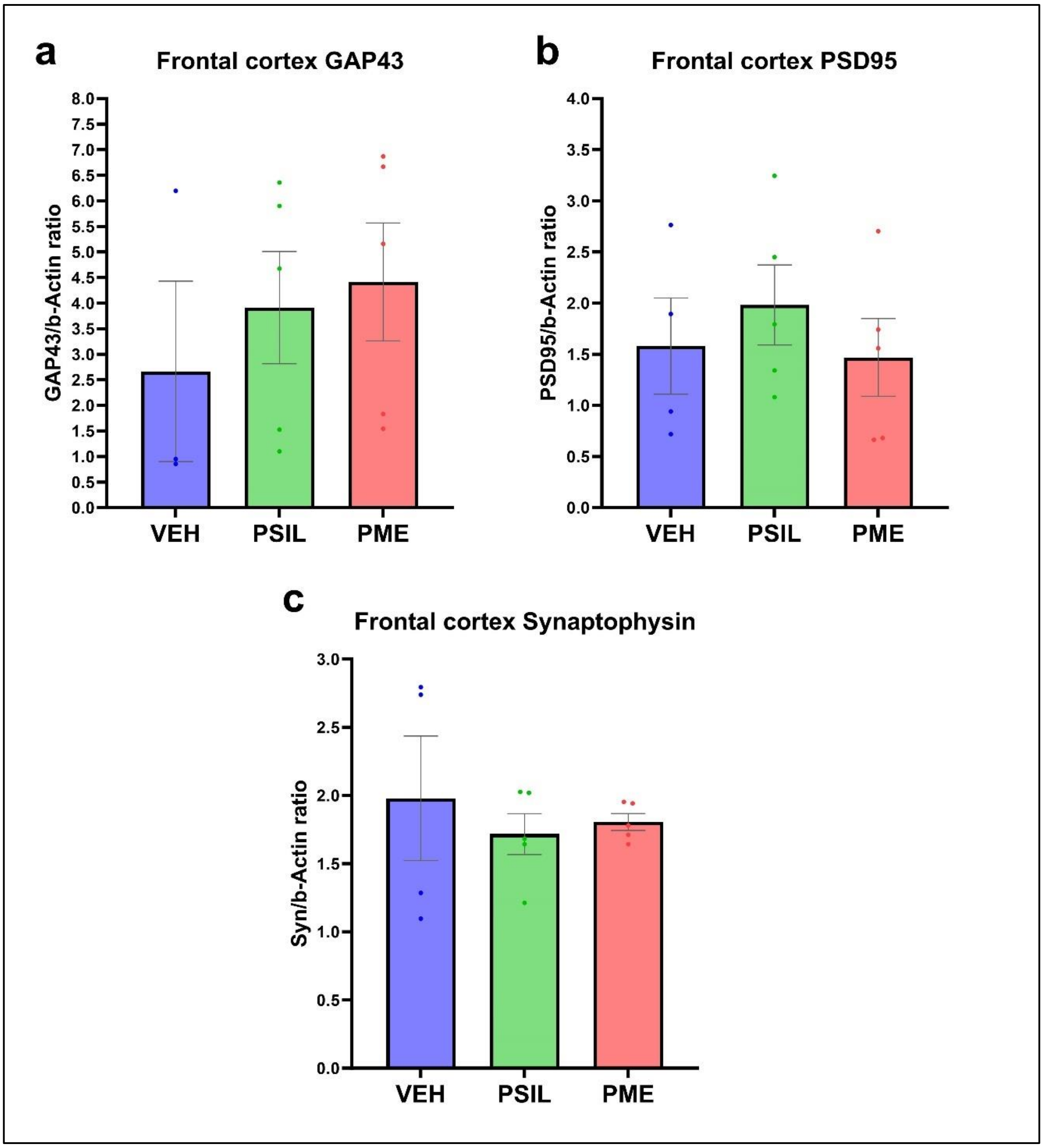

Fig. S11: 3 days post treatment of either VEH, PSIL, or PME Frontal Cortex synaptic protein increase across 3 synaptic proteins of (a) GAP43, (b) PSD95, (c) Synaptophysin (n = 4). One-way ANOVA, Dunnett's multiple comparisons post hoc test. Error bars represent SEM.

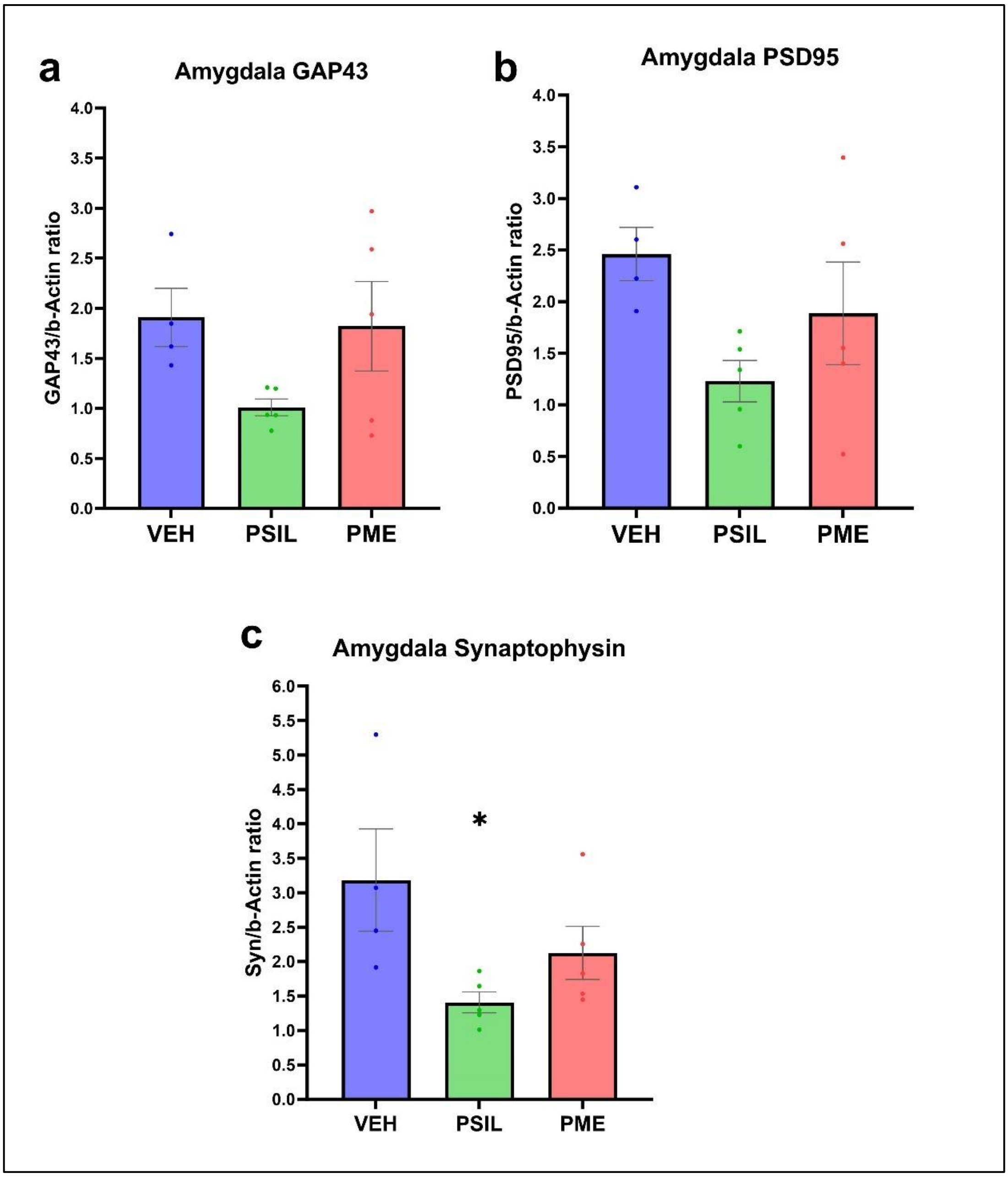

Fig. S12: 3 days post treatment of either VEH, PSIL, or PME Amygdala synaptic protein increase across 3 synaptic proteins of (a) GAP43, (b) PSD95, (c) Synaptophysin (n = 4). One-way ANOVA, Dunnett's multiple comparisons post hoc test. Error bars represent SEM.

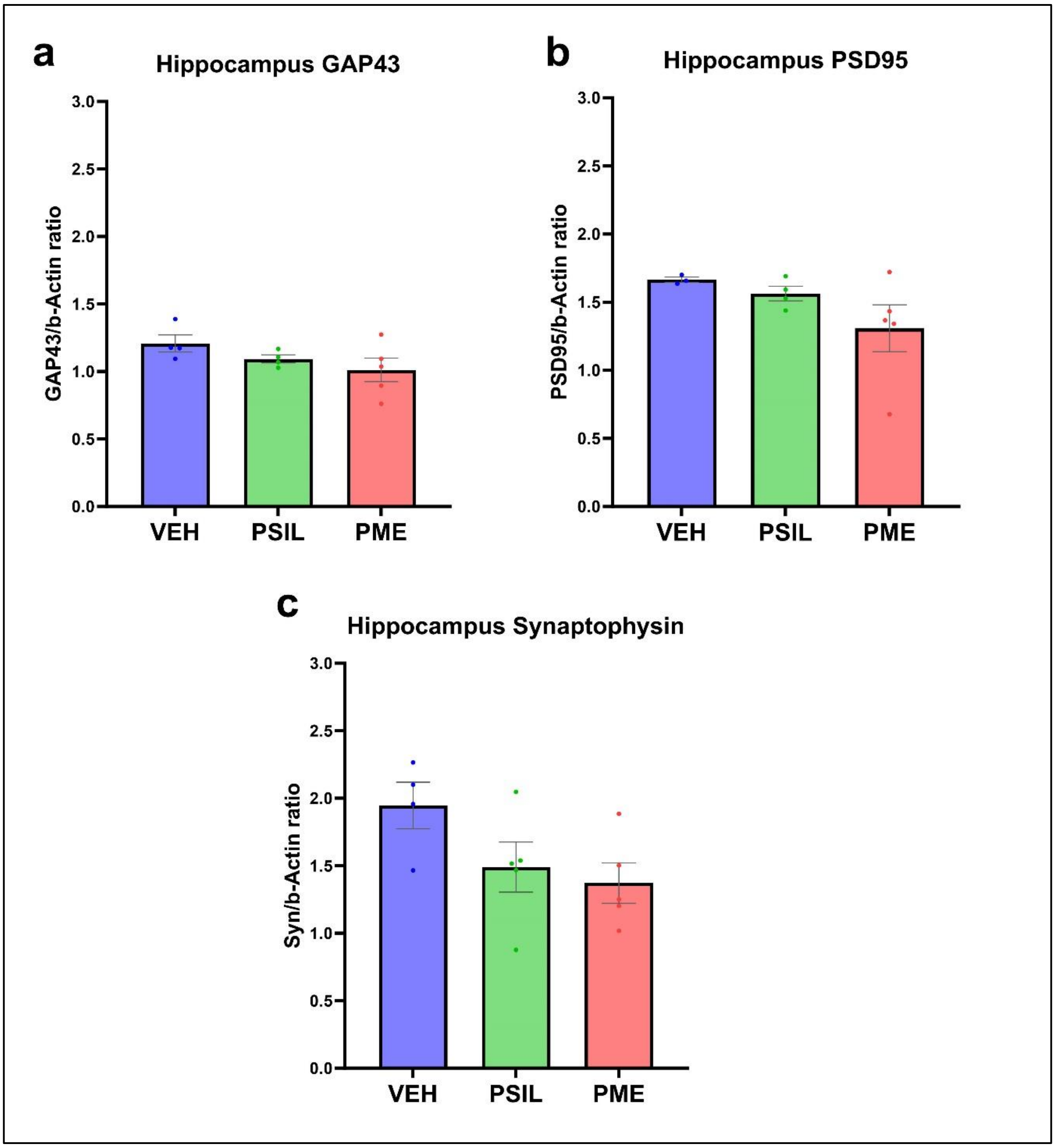

Fig. S13: 3 days post treatment of either VEH, PSIL, or PME Hippocampus synaptic protein increase across 3 synaptic proteins of (a) GAP43, (b) PSD95, (c) Synaptophysin (n = 4). One-way ANOVA, Dunnett's multiple comparisons post hoc test. Error bars represent SEM.

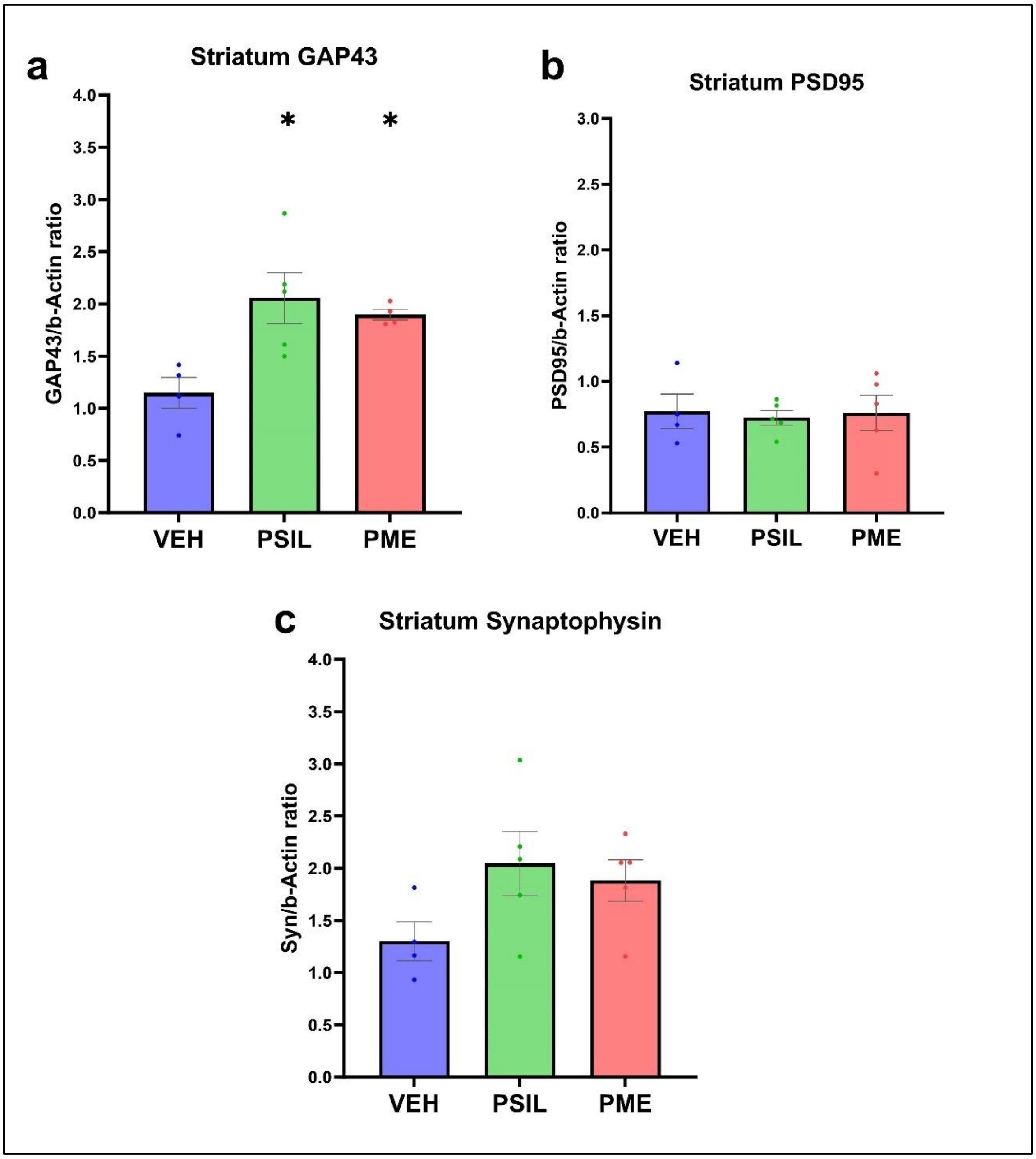

Fig. S14: 3 days post treatment of either VEH, PSIL, or PME Striatum synaptic protein increase across 3 synaptic proteins of (a) GAP43, (b) PSD95, (c) Synaptophysin (n = 4). One-way ANOVA, Dunnett's multiple comparisons post hoc test. Compared to VEH, * $p < 0.05$. Error bars represent SEM.

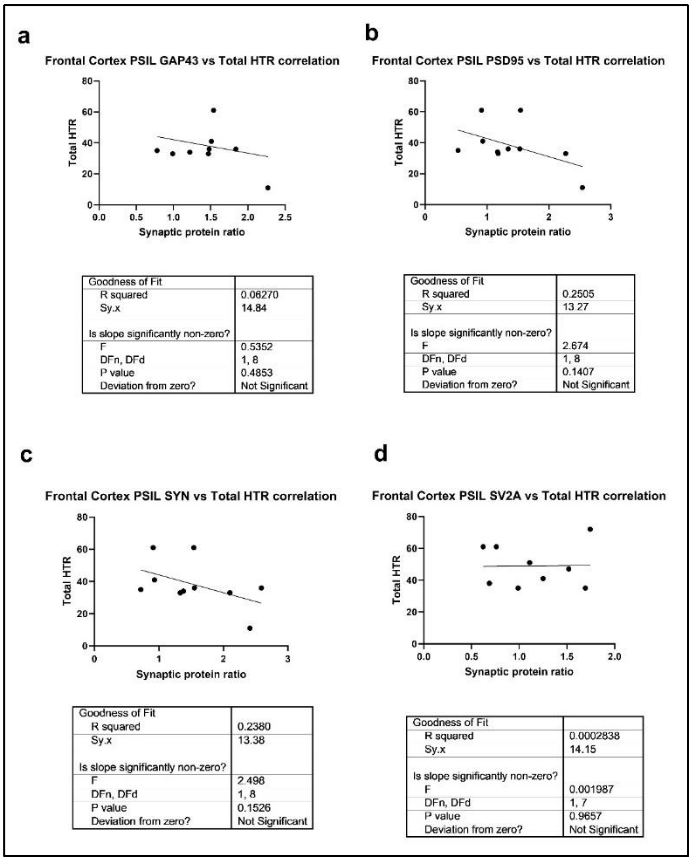

Fig. S15: Simple regression correlation test between Total HTR induced by PSIL and the synaptic proteins (a) GAP43, (b) PSD95, (c) Synaptophysin, (d) SV2A in the Frontal Cortex (n = 10).

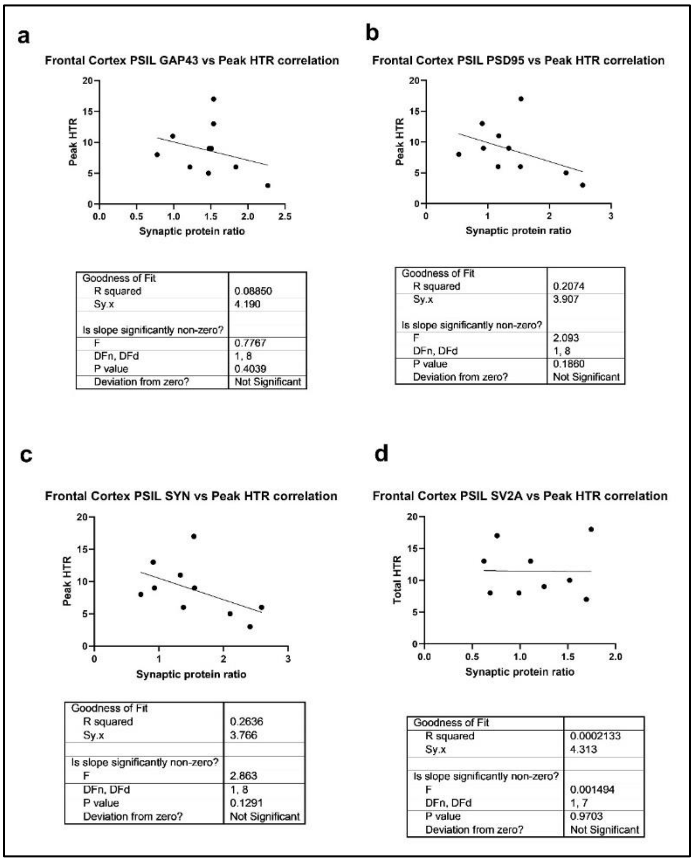

Fig. S16: Simple regression correlation test between Peak HTR induced by PSIL and the synaptic proteins (a) GAP43, (b) PSD95, (c) Synaptophysin, (d) SV2A in the Frontal Cortex (n = 10).

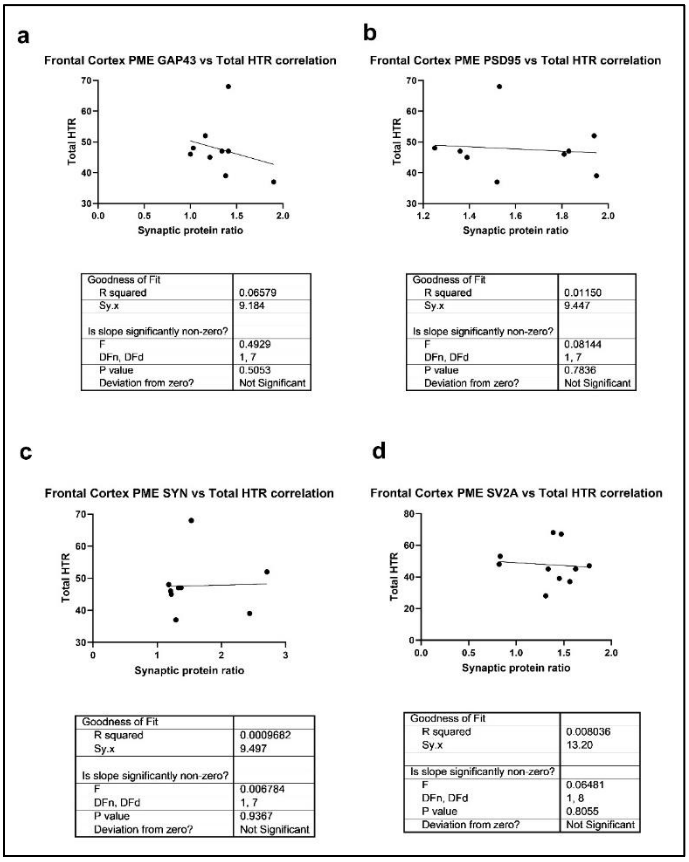

(a)

Goodness of Fit	
R squared	0.06579
Sy.x	9.184
Is slope significantly non-zero?	
F	0.4929
DFn, DFd	1, 7
P value	0.5053
Deviation from zero?	Not Significant

(b)

Goodness of Fit	
R squared	0.01150
Sy.x	9.447
Is slope significantly non-zero?	
F	0.08144
DFn, DFd	1, 7
P value	0.7836
Deviation from zero?	Not Significant

(c)

Goodness of Fit	
R squared	0.0009682
Sy.x	9.497
Is slope significantly non-zero?	
F	0.006784
DFn, DFd	1, 7
P value	0.9367
Deviation from zero?	Not Significant

(d)

Goodness of Fit	
R squared	0.008036
Sy.x	13.20
Is slope significantly non-zero?	
F	0.06481
DFn, DFd	1, 8
P value	0.8055
Deviation from zero?	Not Significant

Fig. S17: Simple regression correlation test between Total HTR induced by PME and the synaptic proteins (a) GAP43, (b) PSD95, (c) Synaptophysin, (d) SV2A in the Frontal Cortex (n = 10).

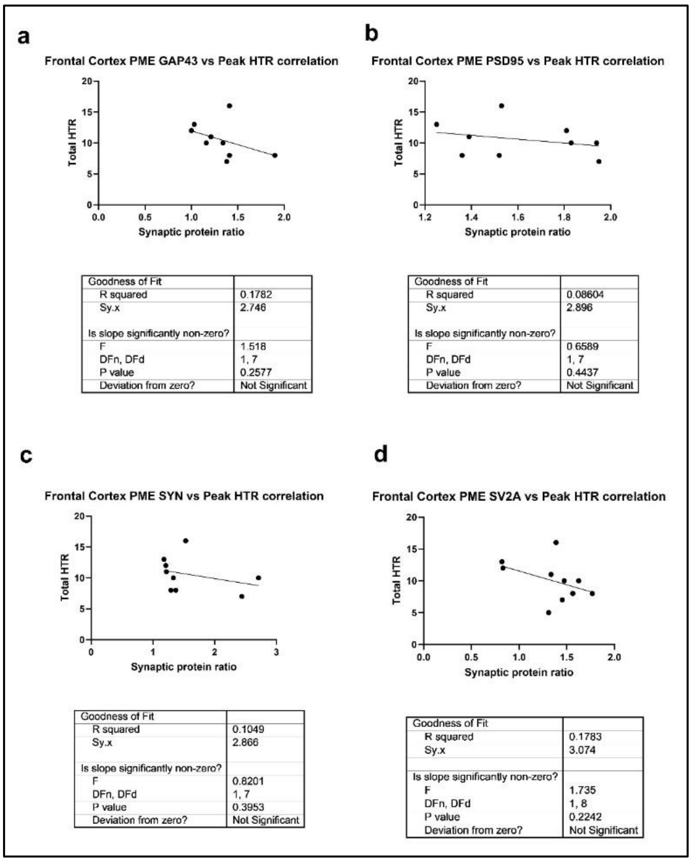

(a)

Goodness of Fit	
R squared	0.1782
Sy.x	2.746
Is slope significantly non-zero?	
F	1.518
DFn, DFd	1, 7
P value	0.2577
Deviation from zero?	Not Significant

(b)

Goodness of Fit	
R squared	0.08604
Sy.x	2.896
Is slope significantly non-zero?	
F	0.6589
DFn, DFd	1, 7
P value	0.4437
Deviation from zero?	Not Significant

(c)

Goodness of Fit	
R squared	0.1049
Sy.x	2.866
Is slope significantly non-zero?	
F	0.8201
DFn, DFd	1, 7
P value	0.3953
Deviation from zero?	Not Significant

(d)

Goodness of Fit	
R squared	0.1783
Sy.x	3.074
Is slope significantly non-zero?	
F	1.735
DFn, DFd	1, 8
P value	0.2242
Deviation from zero?	Not Significant

Fig. S18: Simple regression correlation test between Peak HTR induced by PME and the synaptic proteins (a) GAP43, (b) PSD95, (c) Synaptophysin, (d) SV2A in the Frontal Cortex (n = 10).

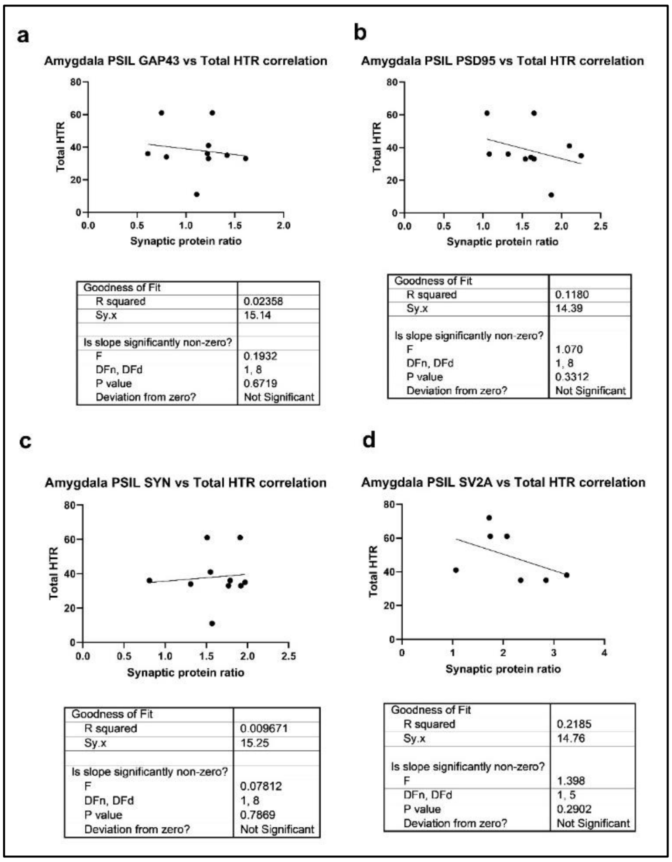

F Fig. S19: Simple regression correlation test between Total HTR induced by PSIL and the synaptic proteins (a) GAP43, (b) PSD95, (c) Synaptophysin, (d) SV2A in the Amygdala (n = 10).

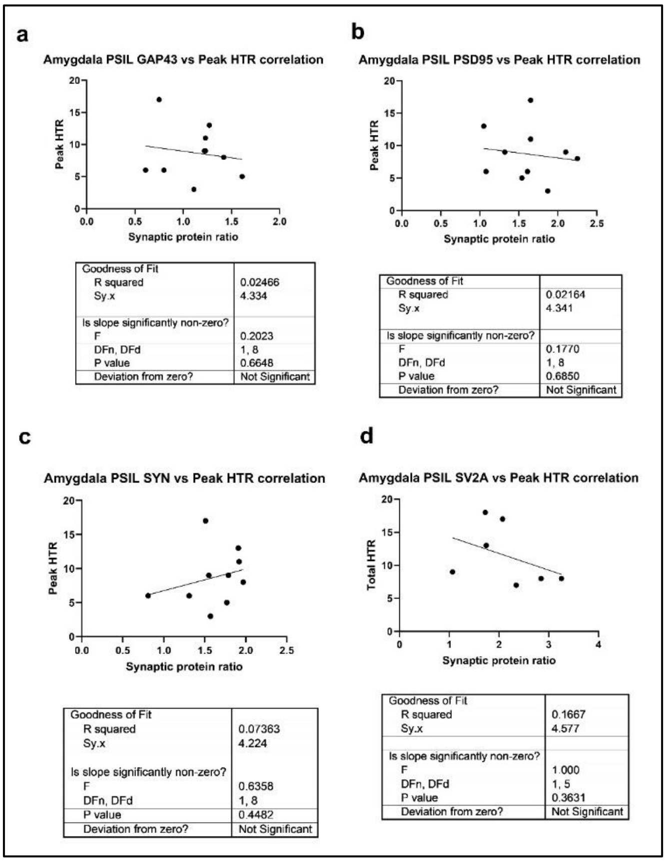

Fig. S20: Simple regression correlation test between Peak HTR induced by PSIL and the synaptic proteins (a) GAP43, (b) PSD95, (c) Synaptophysin, (d) SV2A in the Amygdala (n = 10).

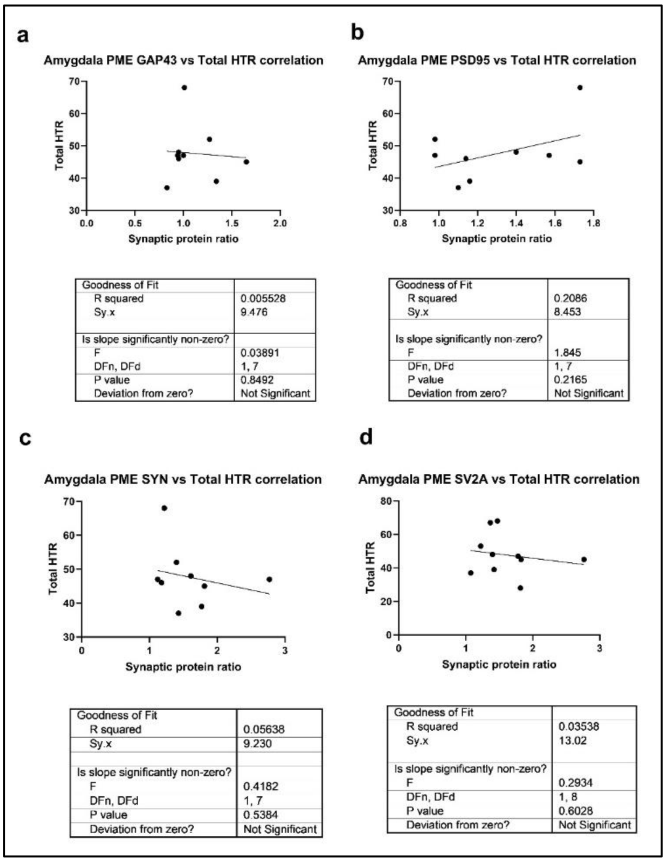

Fig. S21: Simple regression correlation test between Total HTR induced by PME and the synaptic proteins (a) GAP43, (b) PSD95, (c) Synaptophysin, (d) SV2A in the Amygdala (n = 10).

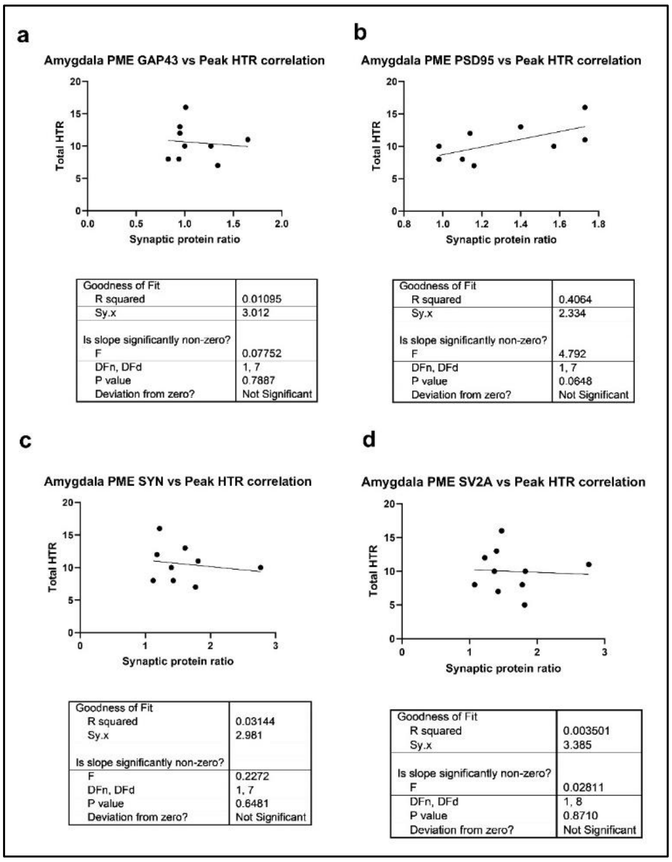

Fig. S22: Simple regression correlation test between Peak HTR induced by PME and the synaptic proteins (a) GAP43, (b) PSD95, (c) Synaptophysin, (d) SV2A in the Amygdala (n = 10).

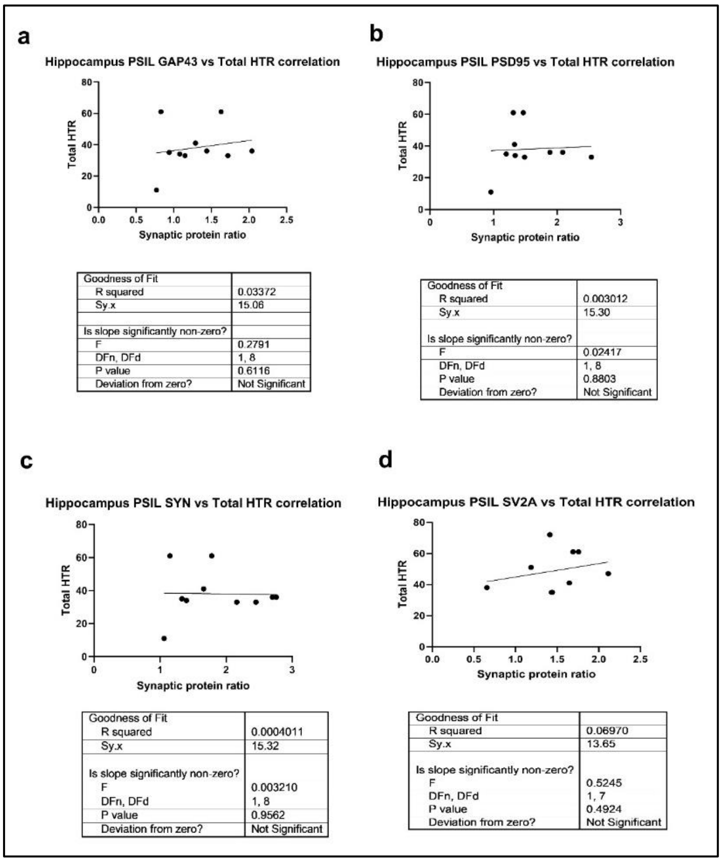

Fig. S23: Simple regression correlation test between Total HTR induced by PSIL and the synaptic proteins (a) GAP43, (b) PSD95, (c) Synaptophysin, (d) SV2A in the Hippocampus (n = 10).

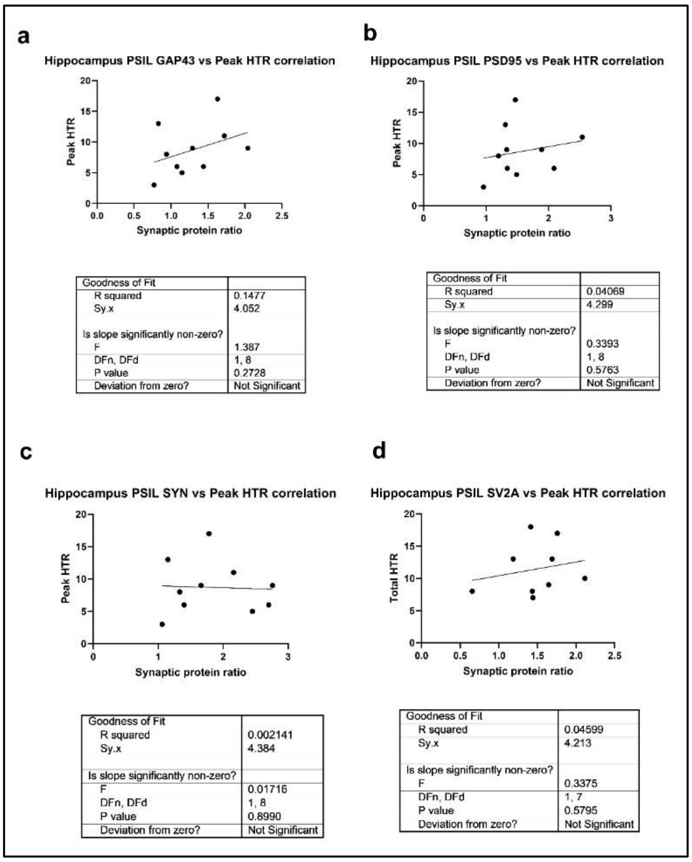

Fig. S24: Simple regression correlation test between Peak HTR induced by PSIL and the synaptic proteins (a) GAP43, (b) PSD95, (c) Synaptophysin, (d) SV2A in the Hippocampus (n = 10).

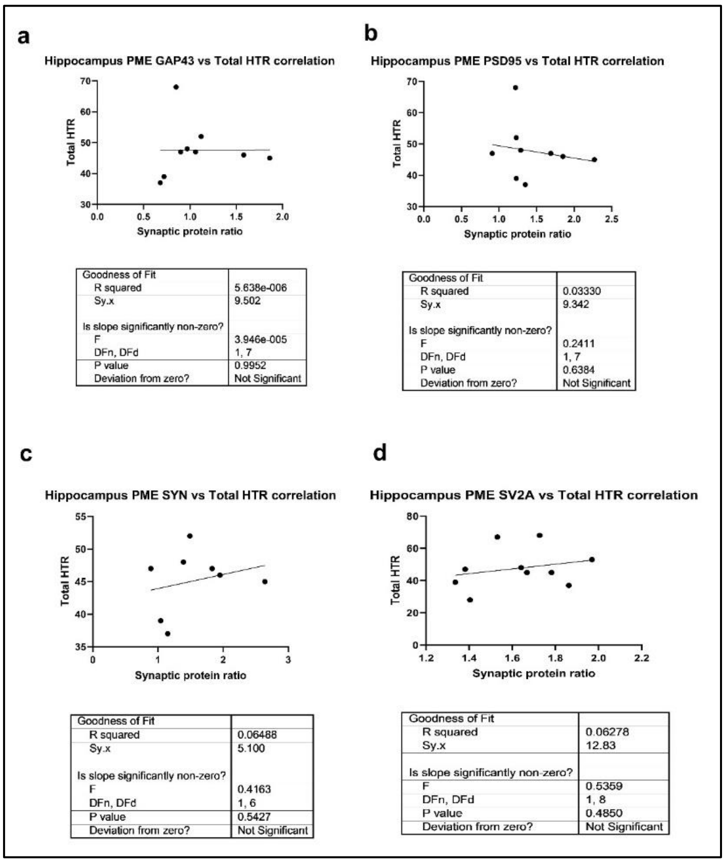

Fig. S25: Simple regression correlation test between Total HTR induced by PME and the synaptic proteins (a) GAP43, (b) PSD95, (c) Synaptophysin, (d) SV2A in the Hippocampus (n = 10).

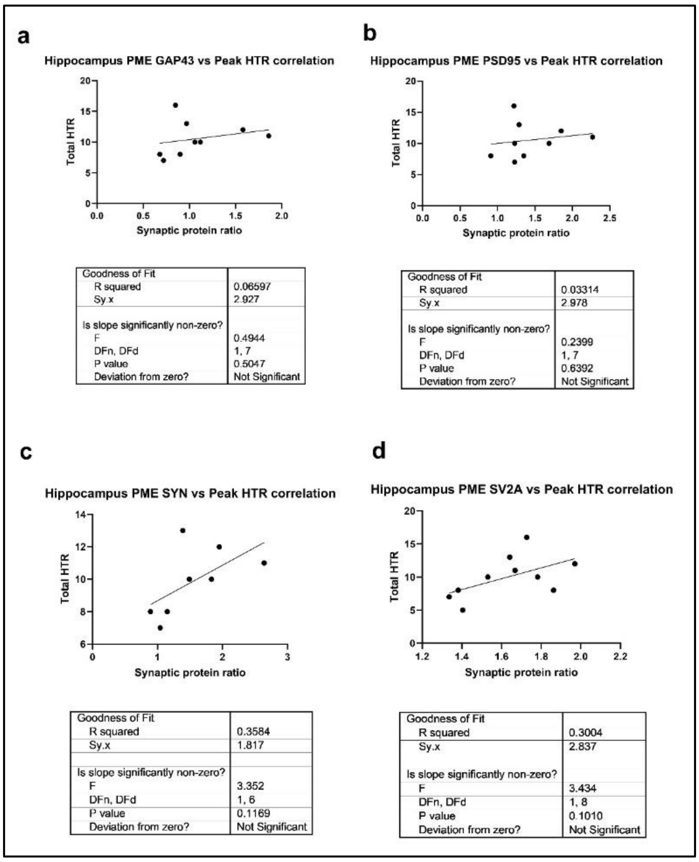

Fig. S26: Simple regression correlation test between Peak HTR induced by PME and the synaptic proteins (a) GAP43, (b) PSD95, (c) Synaptophysin, (d) SV2A in the Hippocampus (n = 10).

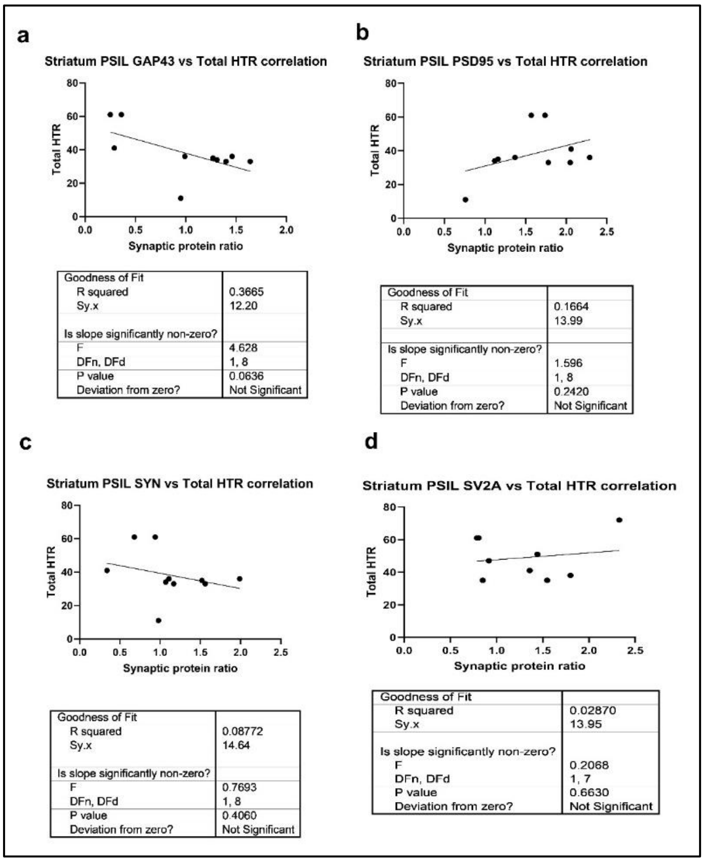

Fig. S27: Simple regression correlation test between Total HTR induced by PSIL and the synaptic proteins (a) GAP43, (b) PSD95, (c) Synaptophysin, (d) SV2A in the Striatum (n = 10).

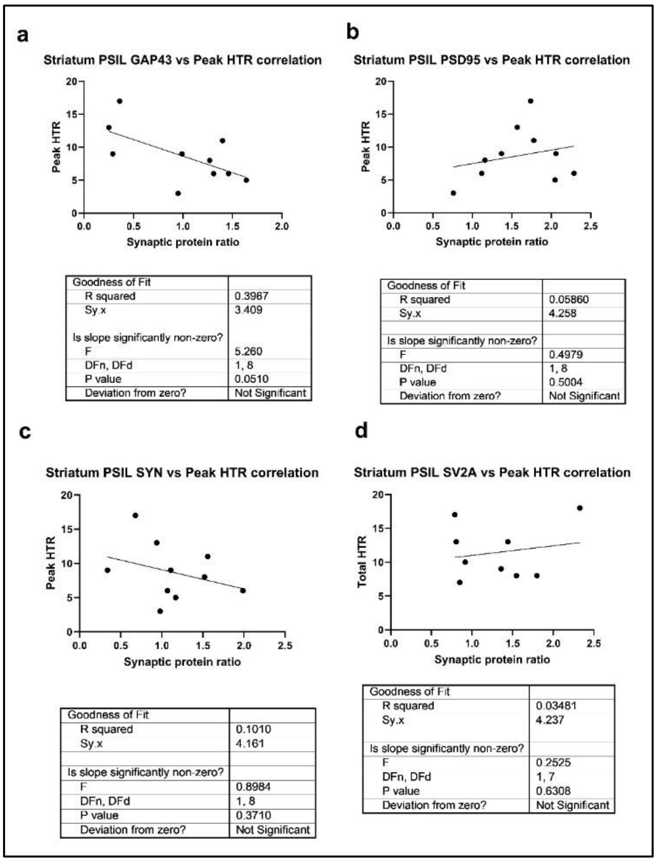

Fig. S28: Simple regression correlation test between Peak HTR induced by PSIL and the synaptic proteins (a) GAP43, (b) PSD95, (c) Synaptophysin, (d) SV2A in the Striatum (n = 10).

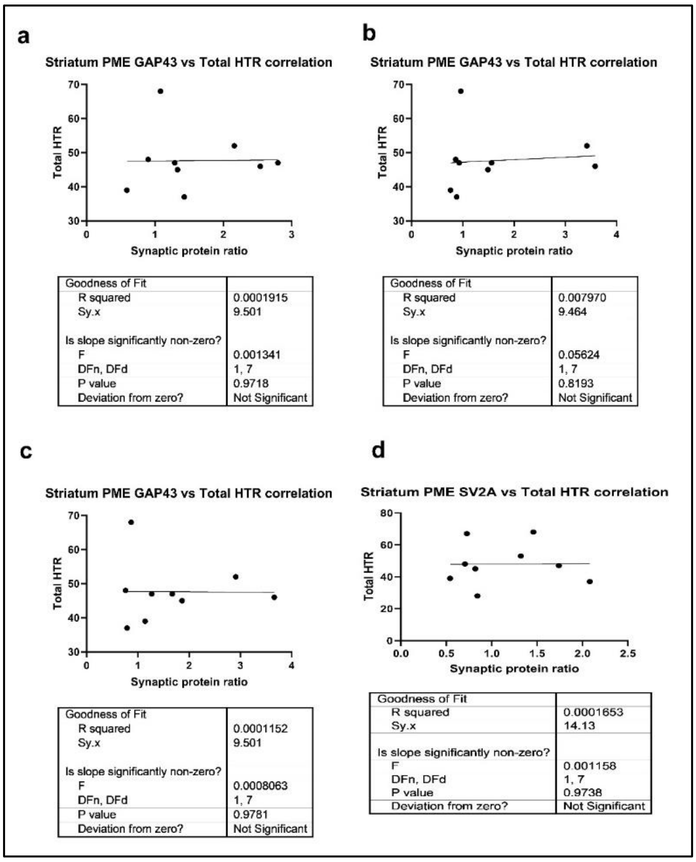

Fig. S29: Simple regression correlation test between Total HTR induced by PME and the synaptic proteins (a) GAP43, (b) PSD95, (c) Synaptophysin, (d) SV2A in the Striatum (n = 10).

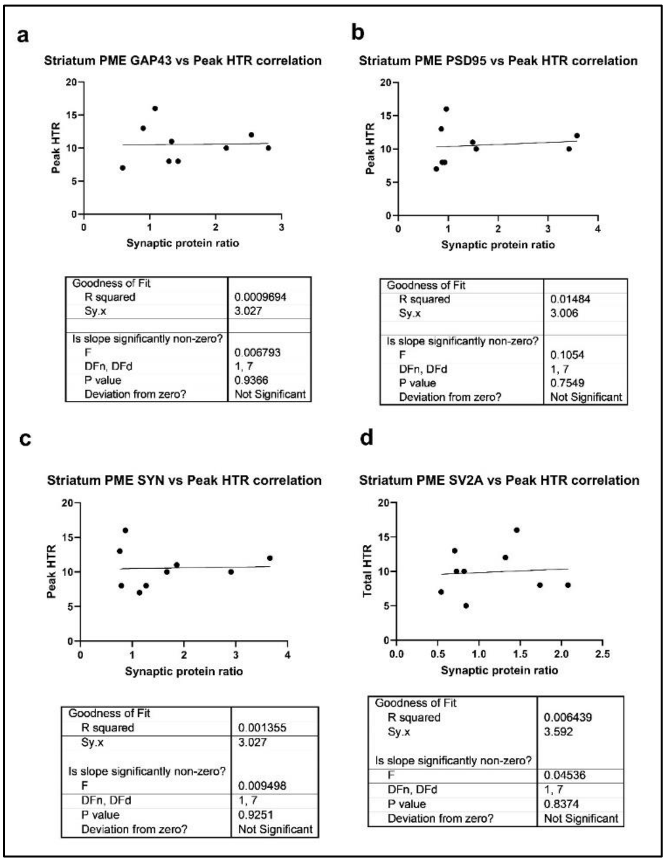

Fig. S30: Simple regression correlation test between Peak HTR induced by PME and the synaptic proteins (a) GAP43, (b) PSD95, (c) Synaptophysin, (d) SV2A in the Striatum (n = 10).

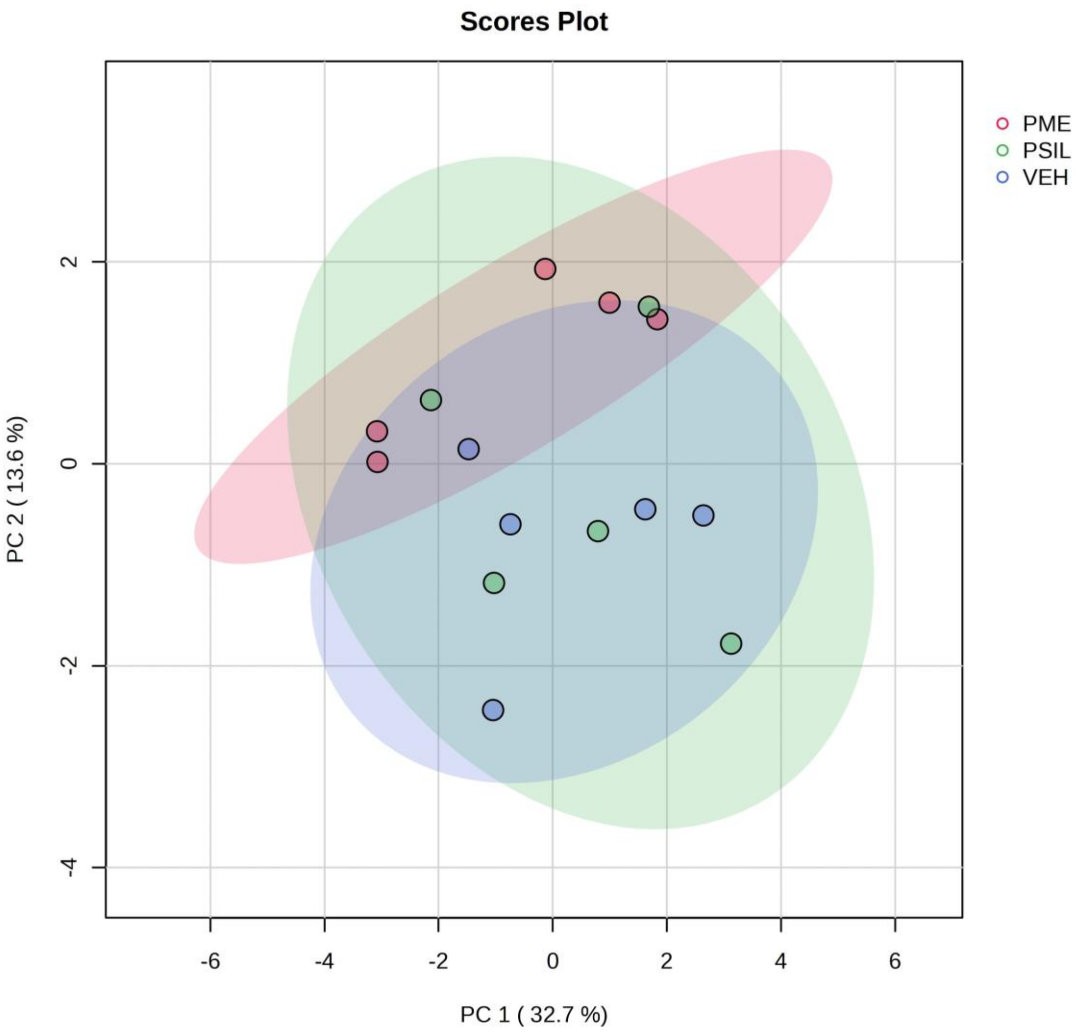

Fig. S31: Principal component analysis (PCA) of treatment groups in the study. Data points represent individual samples for the following groups: psychedelic mushroom extract (PME, red circles), psilocybin (green circles), and vehicle control (blue circles). PCA reveals overlap between all three groups with no clear clustering or separation.

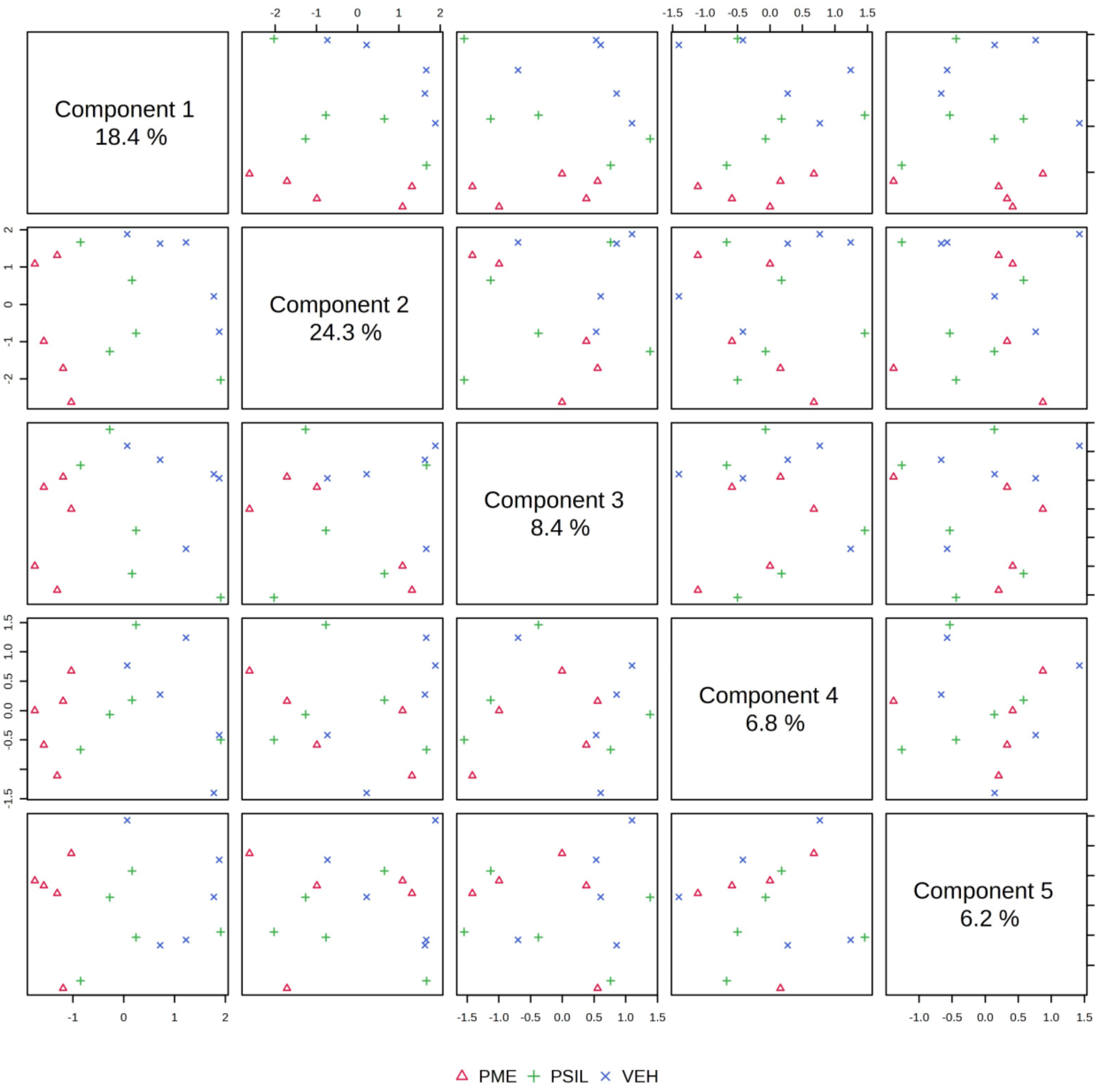

Fig. S32: Partial least squares discriminant analysis (PLSDA) scores plot of metabolomics data from three experimental groups. Data points represent individual samples for the following groups: psychedelic mushroom extract (PME, red triangles), psilocybin (green plus signs), and vehicle control (blue X's). The first two principal components from the PLSDA model explain 42.7% of the total variance (PC1: 18.4%; PC2: 24.3%). The additional principal components each accounted for <10% of the variance and were excluded from the analysis

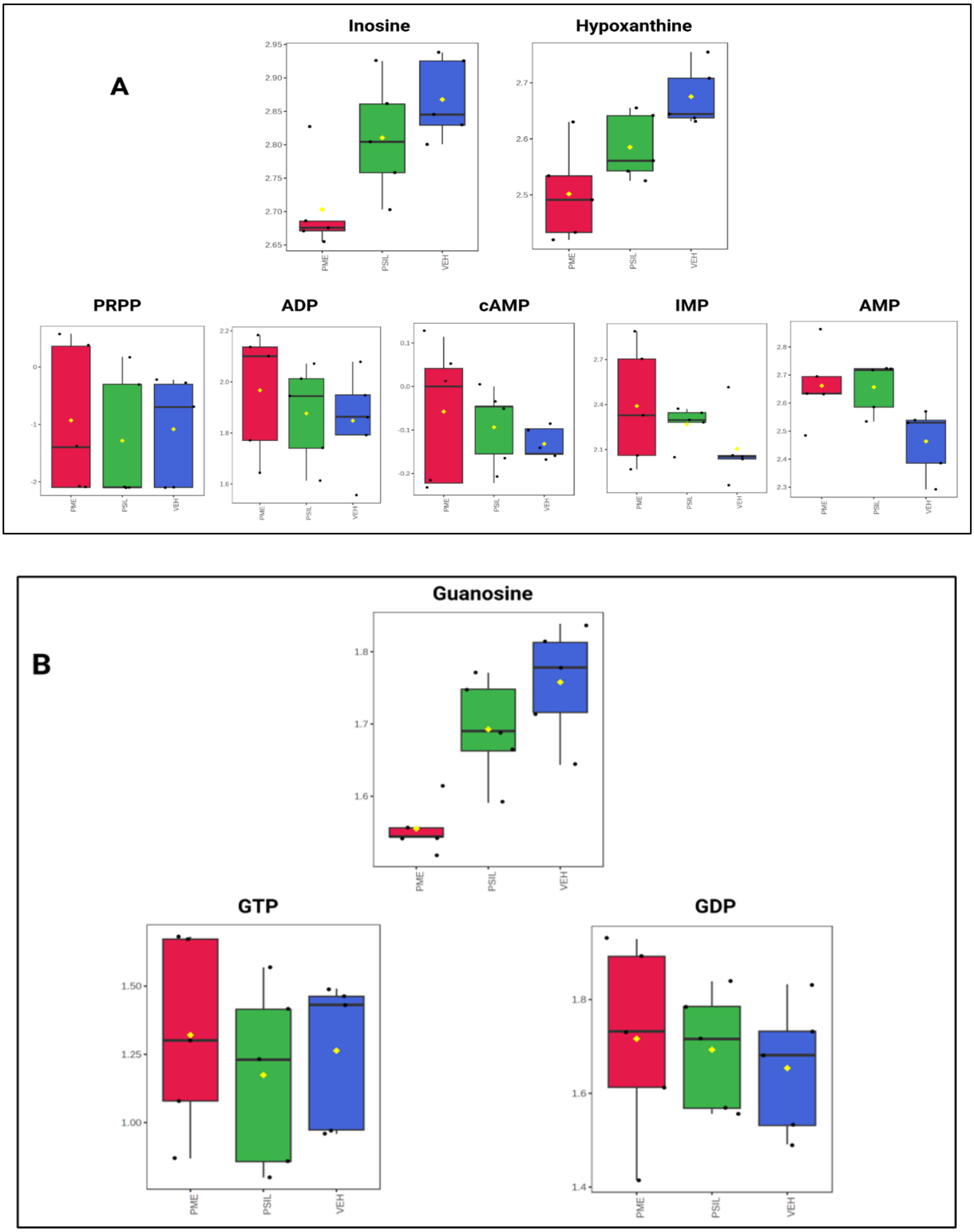

Fig S33: Normalized metabolite expression levels (log transformation) in VEH, PSIL and PME. A. Inosine and hypoxanthine show a progressive decline from VEH via PSIL to PME, while the phosphorilated forms (PRPP, ADP, cAMP, IMP and AMP), are upregulated in the opposite direction.

B. Similarly, guanosine is downregulated in PSIL and PME compared to VEH, while GDP and GTP are gradually upregulated

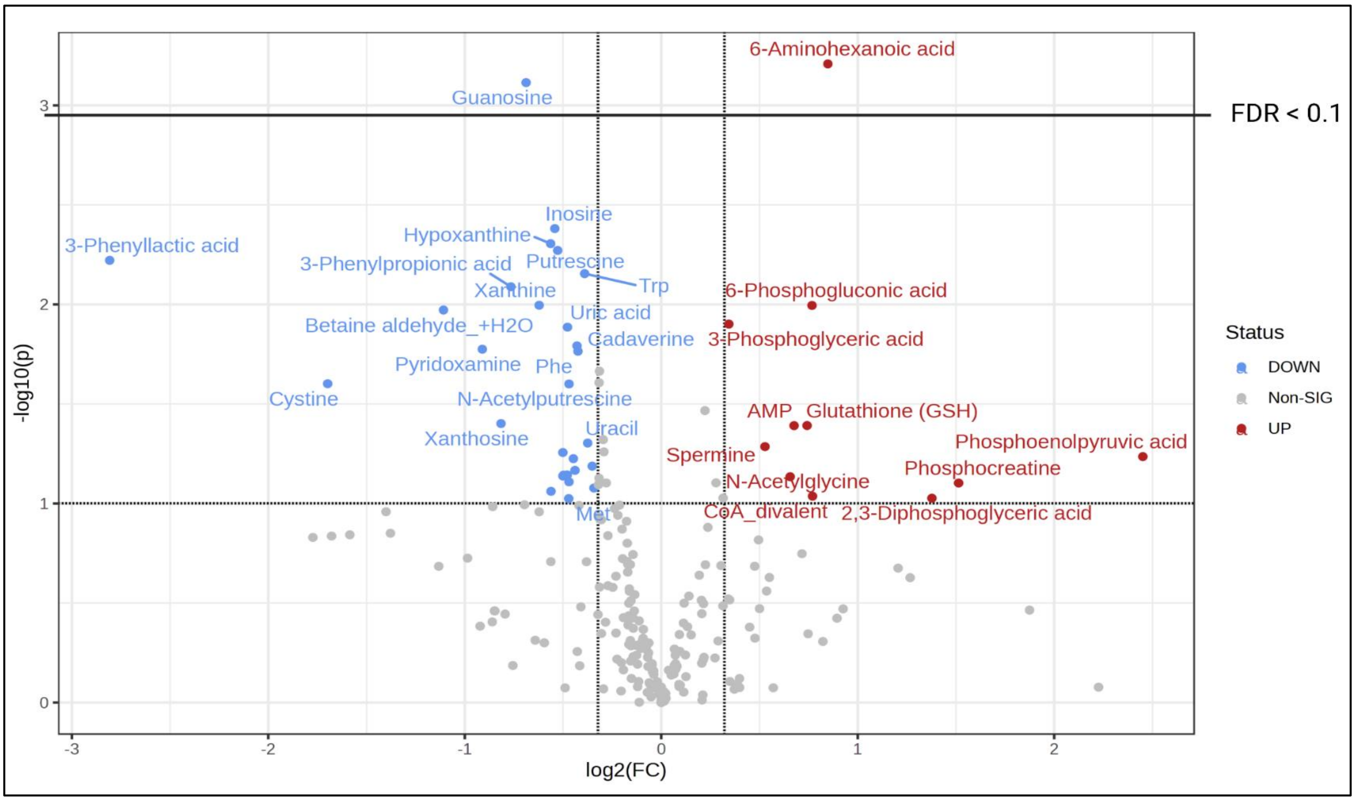

Fig S34: Volcano plot for PME vs. VEH. Red represents metabolites upregulated in PME and blue metabolites are down regulated ($p < 0.05$). Guanosine and 6-aminohexanoic acid withstand FDR correction (FDR < 0.1).

A

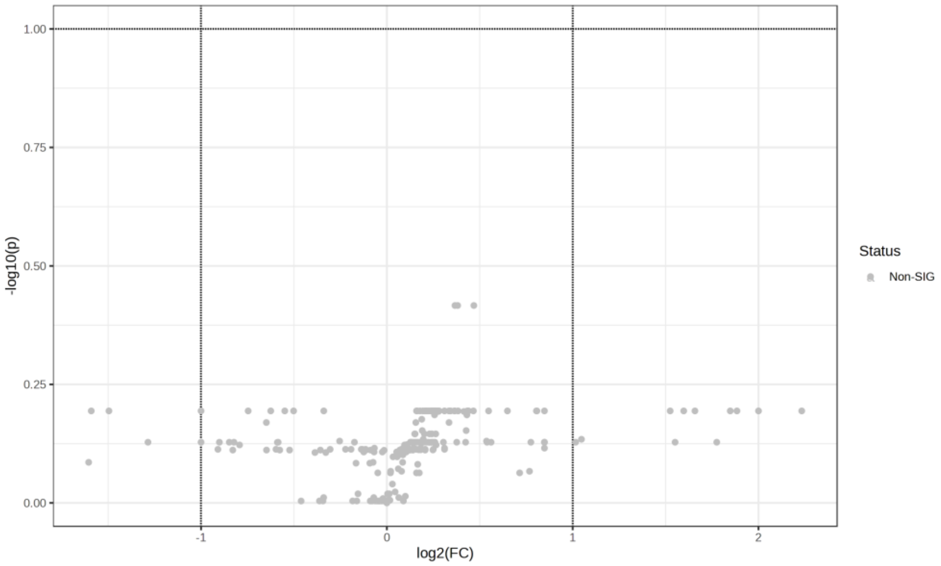

B

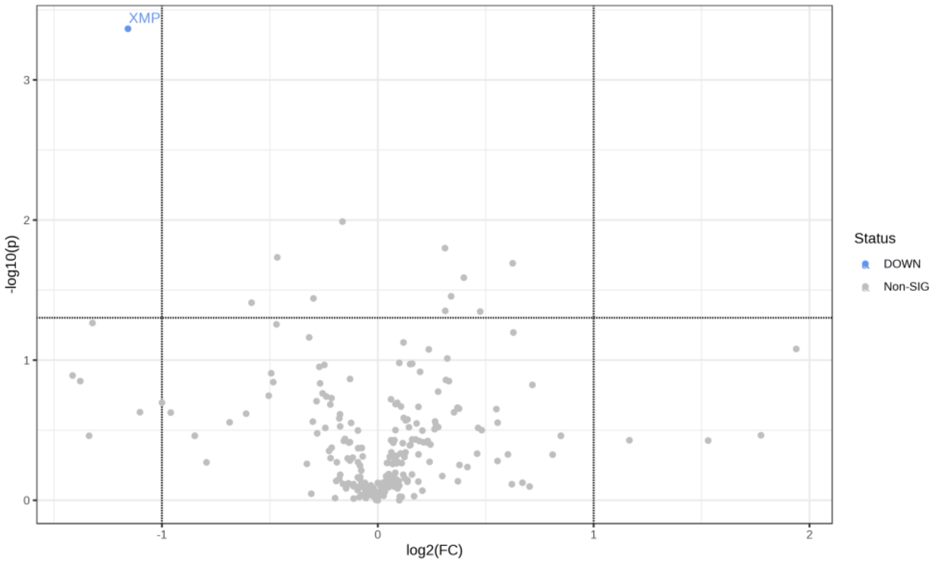

Fig. S35: Fold change comparisons of metabolite levels between additional experimental groups. The volcano plots show pairwise comparisons of psychedelic mushroom extract (PME) versus psilocybin (PSIL) (Plot A) and PSIL versus vehicle control (Plot B). No metabolites exhibited significantly differential expression between either comparison pair after false discovery rate (FDR) adjustment at $p < 0.1$.

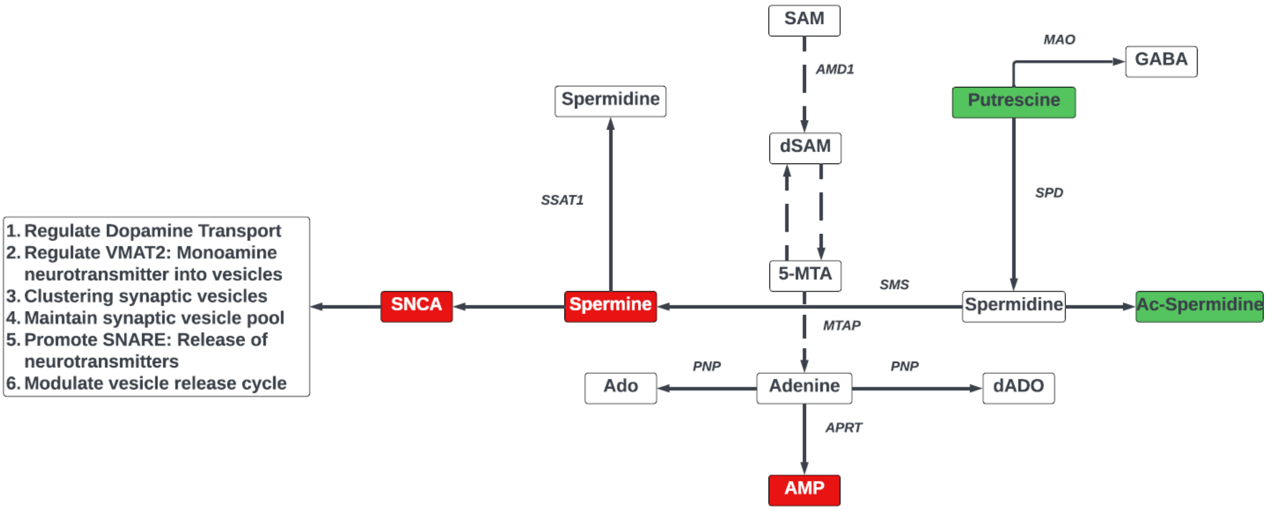

Fig. S36: Schematic representation of the polyamine pathway which is linked to purine metabolism via methylthioadenosine phosphorylase (MTAP). Levels of putrescine, spermine, and their ratio as well as GABA were significantly increased in PME versus vehicle but unaffected in PSIL versus vehicle. The purine salvage enzyme MTAP was uniquely upregulated in PME compared to PSIL and vehicle. PSIL decreased alpha-synuclein (SNCA) versus vehicle while PME increased SNCA.

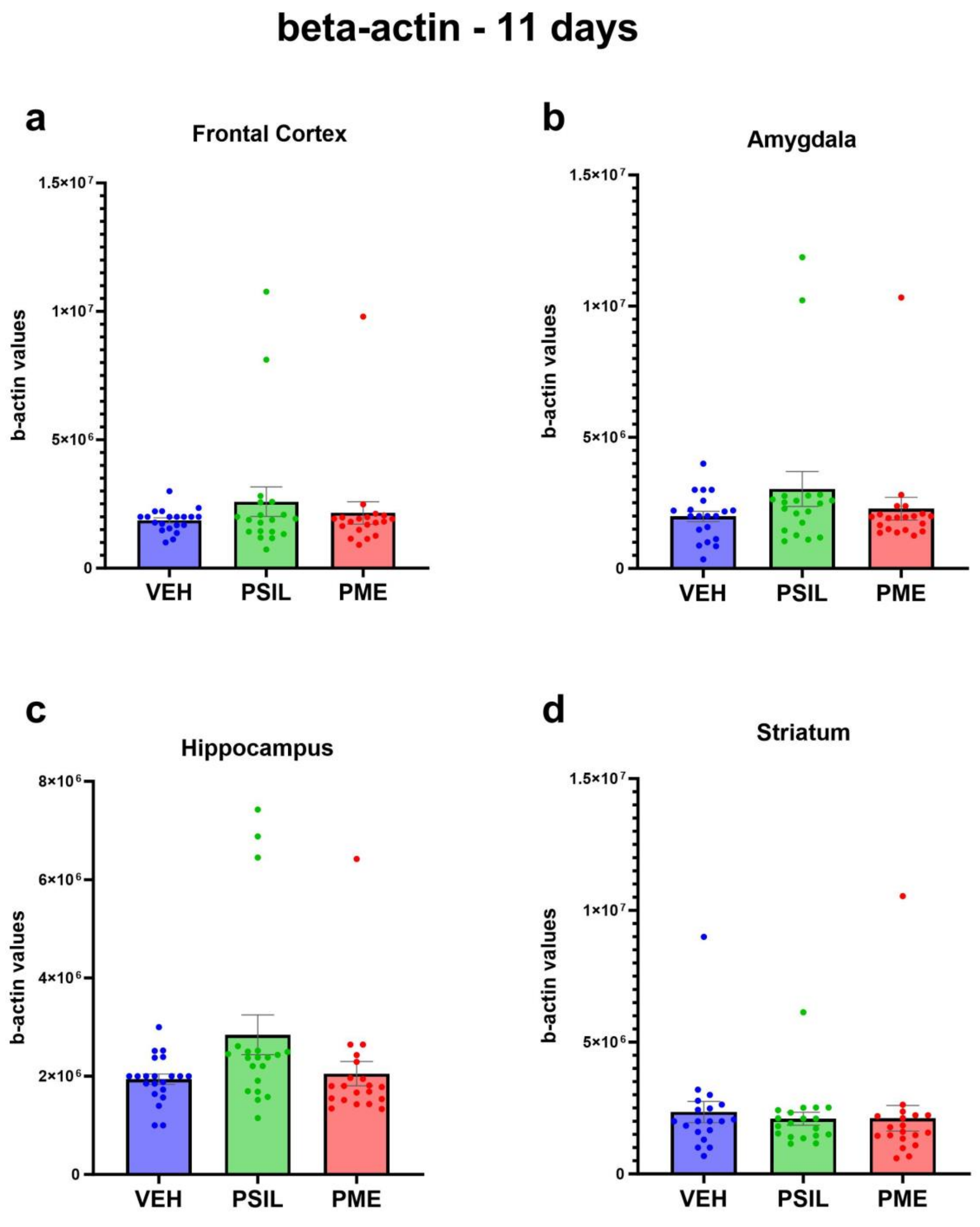

Figure S37. β-actin protein expression levels 11 days post treatment across the brain regions of (a) Frontal Cortex, (b) Amygdala, (c) Hippocampus, (d) Striatum (n = 19-20). One-way ANOVA (a) F (2, 55) = 1.299 p = 0.4581 (b) F (2, 56) = 0.9422 p = 0.2724, (c) F (2, 57) = 1.017 p = 0.1361, (d) F (2, 54) = 0.2078 p = 0.8739; Tukey multiple comparisons post hoc test. Error bars represent SEM.

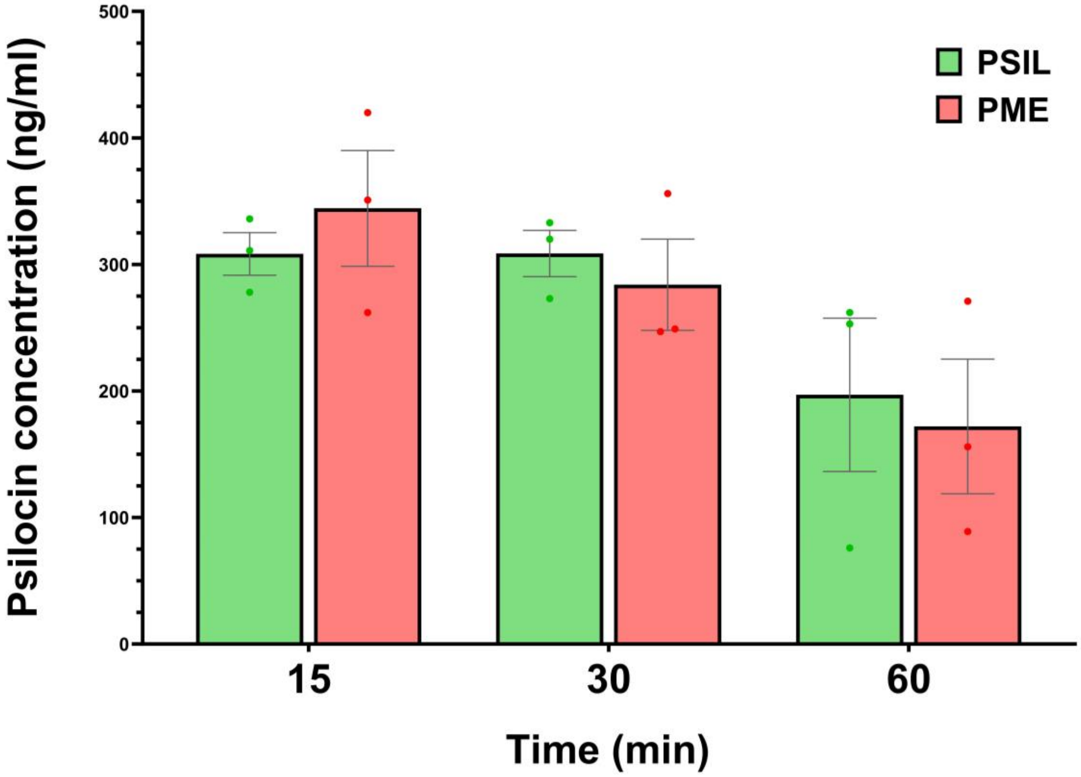

Figure S38: Plasma psilocin levels 15, 30 or 60 minutes after treatment administration in mice administered PSIL (4.4 mg/kg i.p.) or PME (4.4 mg/kg i.p. psilocybin). No differences were statistically significant at any of the three time points (by two way ANOVA – treatment x timepoint and Sidak's post hoc test). N=3 per group.
